# A Novel Antiviral Target Structure Involved in the RNA Binding, Dimerization, and Nuclear Export Functions of the Influenza A Virus Nucleoprotein

**DOI:** 10.1371/journal.ppat.1005062

**Published:** 2015-07-29

**Authors:** Michinori Kakisaka, Yutaka Sasaki, Kazunori Yamada, Yasumitsu Kondoh, Hirokazu Hikono, Hiroyuki Osada, Kentaro Tomii, Takehiko Saito, Yoko Aida

**Affiliations:** 1 Viral Infectious Diseases Unit, RIKEN, Wako, Saitama, Japan; 2 Computational Biology Research Center (CBRC), National Institute of Advanced Industrial Science and Technology (AIST), Koto-ku, Tokyo, Japan; 3 Chemical Biology Group, RIKEN CSRS, Wako, Saitama, Japan; 4 Influenza and Prion Disease Research Center, National Institute of Animal Health, National Agriculture and Food Research Organization (NARO), Tsukuba, Ibaraki, Japan; St. Jude Children's Research Hospital, UNITED STATES

## Abstract

Developing antiviral therapies for influenza A virus (IAV) infection is an ongoing process because of the rapid rate of antigenic mutation and the emergence of drug-resistant viruses. The ideal strategy is to develop drugs that target well-conserved, functionally restricted, and unique surface structures without affecting host cell function. We recently identified the antiviral compound, RK424, by screening a library of 50,000 compounds using cell-based infection assays. RK424 showed potent antiviral activity against many different subtypes of IAV *in vitro* and partially protected mice from a lethal dose of A/WSN/1933 (H1N1) virus *in vivo*. Here, we show that RK424 inhibits viral ribonucleoprotein complex (vRNP) activity, causing the viral nucleoprotein (NP) to accumulate in the cell nucleus. *In silico* docking analysis revealed that RK424 bound to a small pocket in the viral NP. This pocket was surrounded by three functionally important domains: the RNA binding groove, the NP dimer interface, and nuclear export signal (NES) 3, indicating that it may be involved in the RNA binding, oligomerization, and nuclear export functions of NP. The accuracy of this binding model was confirmed in a NP-RK424 binding assay incorporating photo-cross-linked RK424 affinity beads and in a plaque assay evaluating the structure-activity relationship of RK424. Surface plasmon resonance (SPR) and pull-down assays showed that RK424 inhibited both the NP-RNA and NP-NP interactions, whereas size exclusion chromatography showed that RK424 disrupted viral RNA-induced NP oligomerization. In addition, *in vitro* nuclear export assays confirmed that RK424 inhibited nuclear export of NP. The amino acid residues comprising the NP pocket play a crucial role in viral replication and are highly conserved in more than 7,000 NP sequences from avian, human, and swine influenza viruses. Furthermore, we found that the NP pocket has a surface structure different from that of the pocket in host molecules. Taken together, these results describe a promising new approach to developing influenza virus drugs that target a novel pocket structure within NP.

## Author Summary

Influenza A virus nucleoprotein (NP) is a highly conserved multifunctional protein that plays an essential role in infection by all subtypes of influenza A virus, making it an attractive target for new antiviral drugs. NP regulates viral polymerase activity and transport of the viral genome into/from the host cell nucleus by forming the viral ribonucleoprotein complex (vRNP). Because NP regulates replication and transcription of the viral genome in addition to its role in nuclear export (all of which are essential for the production of viral progeny), it is a promising drug target. Here, we used the antiviral compound RK424 to identify a novel pocket structure within NP. This structure encompassed three different functional domains that are involved in the above-mentioned replication steps. RK424 inhibits viral genome replication/transcription and nuclear export of NP by destabilizing the NP oligomer and inhibiting the binding of chromosome region maintenance 1 (CRM1) to NP via nuclear export signal (NES) 3, which is located in close proximity to the NP pocket. Taken together, these findings suggest that this small NP pocket is a novel antiviral target.

## Introduction

Influenza A virus (IAV) causes periodic and widespread epidemics or pandemics, which take the form of respiratory diseases with cold-like symptoms; however, the virus can sometimes cause serious disease with high mortality rates [1]. One of the few options for treating influenza infection is antivirals. Currently, the only approved classes of anti-influenza virus drugs are viral M2 ion channel inhibitors and neuraminidase (NA) inhibitors [2,3]. Although these drugs can be effective for treating influenza infection, the emergence of drug-resistant viral strains is a serious problem [4,5,6]. It is for this reason that M2 ion channel blockers are no longer used clinically to treat circulating IAV strains. Moreover, NA inhibitor-resistant viruses are beginning to emerge. In addition to these conventional antivirals, a novel antiviral drug, T705 (favipiravir), was approved by the Japanese Ministry of Health. T705 is administered as a prodrug, which is metabolized to its active form, T-705-4-ribofuranosyl-5-triphosphate (T-705RTP), inside the cell. T-705RTP is a purine analog that selectively inhibits the influenza virus RNA-dependent RNA polymerase (RdRp) [7]. However, because T-705 is a ribonucleic analog, adverse effects may occur (indeed, such effects are observed after ribavirin treatment) [8]. Indeed, the use of this drug is limited to the treatment of newly-emergent influenza viruses that are resistant to current antivirals; therefore, NA inhibitors are the only drugs currently used to treat the majority of influenza virus infections. Thus, there is an urgent need for new antiviral drugs with novel mechanisms of action.

Recently, the viral nucleoprotein (NP) has attracted interest as a target for new antiviral drugs. NP is the major component of the viral ribonucleoprotein complex (vRNP), which comprises three polymerase subunit proteins (PB2, PB1, and PA) and viral RNA (vRNA) [9]. The vRNP is maintained by a double-helical structure derived from a NP homo-oligomer coupled to the vRNA [10]. This double-helical structure is associated with vRNP functions such as viral replication, nuclear export, and genome packaging. The NPs within the vRNP complex oligomerize via two interaction forces. One comprises an intra-strand interaction through which the tail loop (amino acid (aa) residues 402–428) is inserted into the neighboring NP tail loop pocket [11]. The other comprises an inter-strand interaction between two NP strands with opposite-polarity. The mechanism underlying the interaction between the two NP strands is still not clear; however, two dimer interfaces, the helix-turn-helix motif (aa 149–167) and the C-terminal region (aa 482–498), of NP are involved in this process [12]. After the influenza virus is adsorbed and internalized into the host cell, the vRNP complex is released from the endosome into the cytoplasm from where it is imported into the nucleus. Following virus replication in the nucleus, the vRNP is exported back into the cytoplasm and moves to the cell membrane. During this step, the C- and N-terminal domains of nonstructural protein 2/nuclear export protein (NS2/NEP) mediate the complexation of vRNP-matrix protein 1 (M1) and chromosome region maintenance 1 (CRM1), respectively, to export the vRNP from the nucleus to the cytoplasm [13,14,15,16,17]. In addition, NP is exported from the nucleus via the NES. NP contains functional NESs at aa 24–49 (NES1), 183–197 (NES2), and 248–274 (NES3) [18,19]. Although nuclear export via NES1 and NES2 is CRM-independent, export via NES3 is CRM1-dependent [18,19]. Binding assays show that NP binds directly to CRM1 and that the cytoplasmic localization of NP is inhibited by leptomycin B (LMB), which inhibits the CRM1-mediated nuclear export of cargo proteins [19, 20]. Thus, the direct interaction of NP with CRM1 may be associated with the nuclear export of the vRNP, because NP is a structural component of the vRNP.

The multiple functions of NP mean that it has attracted interest as a novel target for antiviral drugs [21]. A recent study shows that viral replication is inhibited by small-molecule compounds that target NP, including nuclear localization signal (NLS)-binding compounds (i.e., mycalamide analogs) [22] and NP salt bridge inhibitors (i.e., compound 3) [23]. Mycalamide analogs, which were identified by a chemical array screening method, inhibit influenza virus replication by binding to unconventional NLS within the NP [22]. Furthermore, compound 3 inhibits formation of the NP trimer by disrupting the salt bridge between E339 and R416 within the NP tail loop binding pocket, thereby inhibiting the functional oligomerization of NP [23]. Notably, nucleozin inhibits the formation of higher-order NP oligomers by cross-linking two composition of NP, showing a potent antiviral effect both *in vitro* and *in vivo* [24,25]. These results clearly show that influenza virus NP is a valid target for new antiviral compounds.

We previously screened a chemical library containing 50,000 compounds with diverse structures and identified RK424, a compound with antiviral effects. Here, we showed that RK424 inhibits IVA replication via a mechanism that is different from that of existing antiviral drugs. RK424 bound to a small pocket structure within NP, which is surrounded by three different domains (the RNA binging groove, the NP dimer interface, and NES3), thereby inhibiting the function of all three. Furthermore, the amino acid residues that comprise the NP pocket are well-conserved, and mutations at these residues are functionally restricted. Taken together, the results of the present study show that the newly-identified NP pocket is a promising target for antiviral drugs that inhibit the multiple functions of NP by simultaneously disrupting the function of three different functional domains.

## Results

### RK424 shows antiviral activity both *in vitro* and *in vivo*

**Fig 1:**
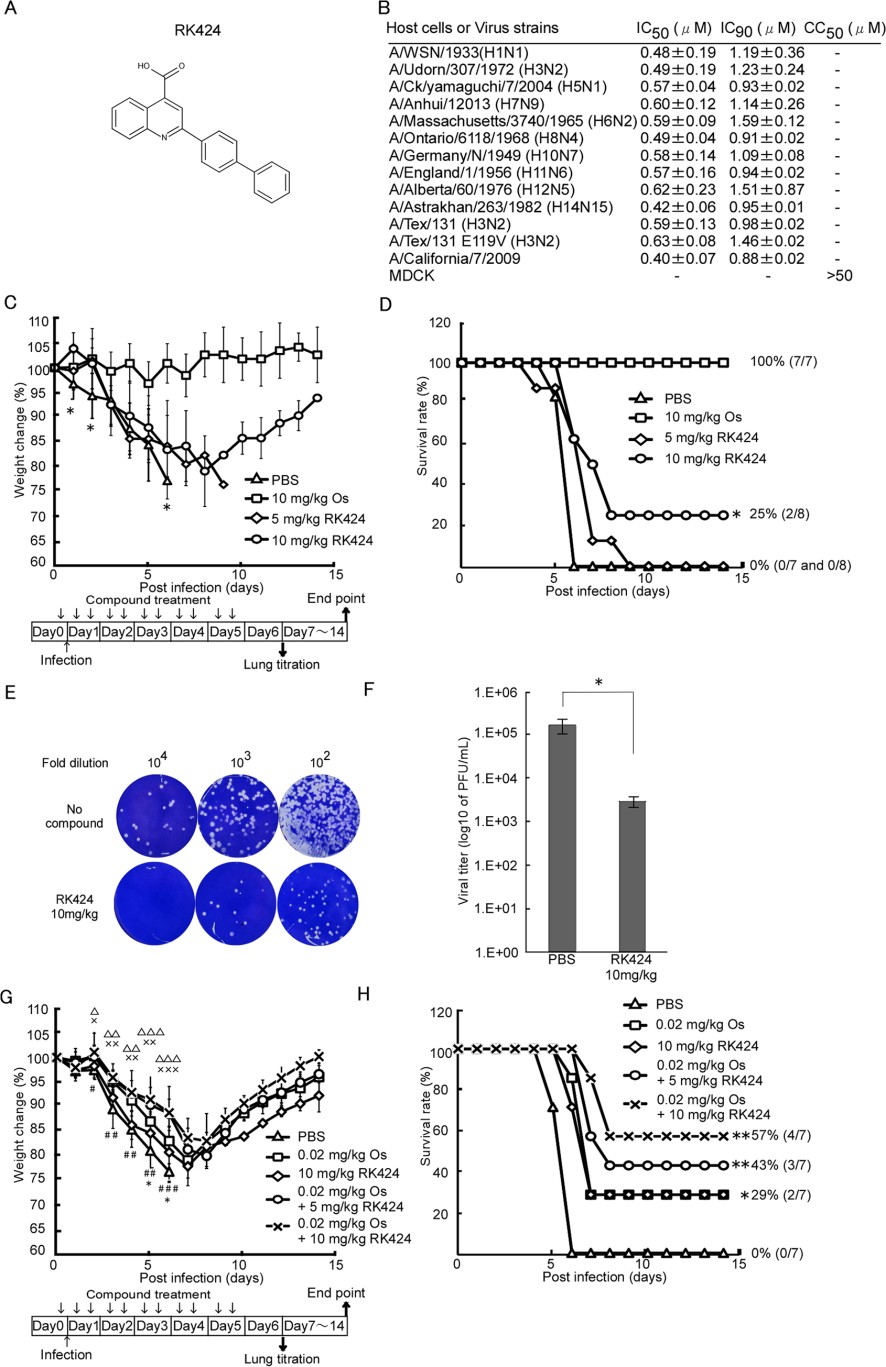
RK424 shows antiviral activity both *in vitro* and *in vivo*. (A) Chemical structure of the RK424. (B) The effects of RK424 on viral replication and MDCK cell viability *in vitro*. The 50% inhibitory concentration (IC_50_) and the 50% cytotoxicity concentration (CC_50_) of RK424 (0–50 μM) were evaluated in a plaque assay and water soluble tetrazolium salt-1 (WST-1) assay, respectively. Data are expressed as the mean ± SD from three samples in each of three independent experiments. (C–H) Inhibitory effect of RK424 *in vivo*. RK424 (5 mg/kg or 10 mg/kg) was intraperitoneally administered to 6-week-old Balb/c mice 2 h prior to virus exposure and then twice per day for 5 days beginning on the day of infection. PBS and oseltamivir phosphate (Os; 10 mg/kg) were used as a negative and positive controls, respectively. Mice were infected intranasally with ten 50% lethal doses (LD_50_) of influenza A/WSN/1933 (H1N1) virus. Eight mice per RK424 treated group and seven mice per both negative and positive control group were tested. The body weight of mice from each group was daily monitored (C) and survival rate were also calculated (D). The data were obtained from a single experiment. In the body weight change data, the symbol (*) indicates a statistically significant difference in mean weight between the 10 mg/kg RK424 treated group and the negative control group at that time point. Values are expressed as the mean ± SD and statistical analysis was performed until day 6 post-infection. *; p<0.05. In the survival curve data, the statistically significant difference between the 10 mg/kg RK424 treated group and the negative control is also indicated. *; p<0.01. Three mice from the 10 mg/kg RK424-treated or PBS-treated group were sacrificed on day 6 post-infection and the lungs were harvested to determine viral titers. Plaque images (E) and the viral lung titers (F) in each group are shown. Values are expressed as the mean ± SD of triplicate assays. *; p<0.05. (G and H) Co-administration of RK424 and Os *in vivo*. K424 (5 mg/kg or 10 mg/kg) and Os (0.02 mg/kg) were intraperitoneally co-administered to 6-week-old Balb/c mice 2 h prior to virus exposure and then twice per day for 5 days beginning on the day of infection. PBS was used as negative control and RK424 (10mg/kg) and Os (0.02mg/kg) were used as monotherapy control, respectively. Eight mice per each group were tested. Body weight change (G) and survival rate (H) of each group were evaluated by a single experiment. In the body weight change data, the symbols (#; 0.02 mg/kg Os, *; 10mg/kg RK424, ×; 0.02mg/kg Os + 5 mg/kg RK424, and △; 0.02 mg/kg Os + 10 mg/kg RK424) indicate a statistically significant difference in mean weight between each compound treated group and the negative control group at that time point. Values are expressed as the mean ± SD and statistical analysis was performed until day 6 post-infection. **#**,*,×,△; p<0.05, **##**,××,△△; p<0.005 and **###**,×××,△△△; p<0.001. In the survival curve data, a statistically significant difference between each compound treated group and the negative control group is also indicated. *; p<0.01 and **; p<0.001.

We previously identified four hit compounds with antiviral activity higher than a 50% inhibitory concentration of 3 μM (inhibitory concentration (IC_50_) ≦3 μM) by screening 50,000 compounds in a Madin-Darby canine kidney (MDCK) cell-based influenza A infection assay [26]. Among these initial hit compounds, RK424 showed the greatest potency (IC_50_ = 0.48±0.19 μM and IC_90_ = 1.19±0.36 μM) against the A/WSN/1933 (H1N1) virus in a plaque assay without inhibiting the growth of MDCK cells (50% cytotoxicity concentration (CC_50_) ≧50 μM) (Fig 1A and 1B). RK424 showed a similar level of antiviral activity against many different subtypes of IAVs including currently circulating A/California/7/2009 virus (H1N1) (IC50 = 0.40±0.07 μM and IC_90_ = 0.88±0.02 μM). Interestingly, RK424 inhibited the replication of the highly pathogenic influenza viruses, A/CX/yamaguchi/7/2004 (H5N1) and A/Anhui/1/2013(H7N9), with IC_50_ values of 0.57±0.04 μM and 0.60±0.12 μM and IC_90_ values of 0.93±0.02 μM and 1.14±0.26 μM, respectively. These results clearly show that RK424 inhibits the replication of IAVs.

We also examined the efficacy of RK424 *in vivo*. Intravenous administration of 1mg/kg RK424 to mice resulted in a free drug maximum concentration (C_free max_) of RK424 (11.4 ng/mL) that was considerably lower than the IC_50_ of RK424 (162.7 ng/mL) in plasma (S1 Table). The dose could not be increased further because levels above 1mg/kg resulted in the precipitation of RK424 from the aqueous vehicle. Besides, the bioavailability of RK424 via the oral route relative to that via the intraperitoneal route was 50% lower and the C_free max_ of RK424 (46.4 ng/mL) did not exceed the IC_50_ of RK424 (162.7 ng/mL) when RK424 was administered at 10 mg/kg via the oral route (S1 Fig and S2 Table). Therefore, mice were intraperitoneally injected with RK424 and then inoculated with ten 50% lethal doses (LD_50_) of A/WSN/1933 (H1N1). Morbidity and mortality were monitored daily by measuring body weight (Fig 1C and 1D). Phosphate buffered saline (PBS)-treated control mice showed severe morbidity and 100% mortality at 6 days post-infection. By contrast, mice treated with RK424 (10 mg/kg) lost less weight and 25% survived until the end of the observation period. RK424 provided high levels of protection against lethal virus infections at early time points (up to 6 days post-infection); however, *in vivo* data showed that RK424 was less effective than oseltamivir phosphate. To confirm the antiviral effects of RK424 *in vivo*, mice were euthanized at 6 days post-infection (the point at which RK424 showed the greatest antiviral effect) and the lungs were harvested for use in a virus titration assay. Lungs from the negative control group showed efficient virus replication whereas those from the RK424-treated group showed a ~2 log reduction in the virus titer (2.9×10^3^ PFU/mL) compared with the titers of the controls (1.6×10^5^ PFU/mL) (Fig 1E and 1F). These results show that RK424 is also effective for the inhibition of viral replication *in vivo*. Moreover, we investigated for synergy in the antiviral effect of RK424 treated with oseltamivir phosphate. First, we determined the most appropriate dose of oseltamivir phosphate (0.02mg/kg Os) for co-administration with RK424 by evaluating the antiviral effect of serial dilutions of oseltamivir phosphate *in vivo* (S2 Fig). Monotherapy groups (10mg/kg RK424 or 0.02 mg/kg Os) showed almost the same body weight changes and survival rates, and 29% of the mice in these groups survived until the end of the observation period. On the other hand, co-administration of RK424 (5 mg/kg or 10 mg/kg) with oseltamivir phosphate (0.02 mg/kg) resulted in less weight loss and a higher survival rate (up to 57%) than the administration of RK424 or oseltamivir phosphate alone. This result further supports the notion that RK424 and oseltamivir phosphate act via different mechanisms and that they elicit a synergistic effect when combined *in vivo*.

However, *in vivo* efficacy with respect to morbidity and mortality was modest compared with the *in vitro* antiviral effect. This discrepancy is perhaps explained by the high plasma protein binding (99.9%) of RK424 (S1 Table), which means that only 0.4% of drug is free from plasma protein. This may have caused a decrease in drug efficacy *in vivo*. Moreover, the volume of distribution (Vd) of RK424 (583 mL/kg) was lower than that of mouse total body water (725 mL/kg) (S2 Table) [27]. These PK parameters may explain its weak pharmacologic efficacy and low tissue absorption *in vivo*. Thus, the plasma protein binding property of RK424 should be optimized to further improve its efficacy *in vivo*.

Taken together, these data suggest that RK424 has antiviral activity both *in vitro* and *in vivo*.

### RK424 inhibits vRNP activity

**Fig 2:**
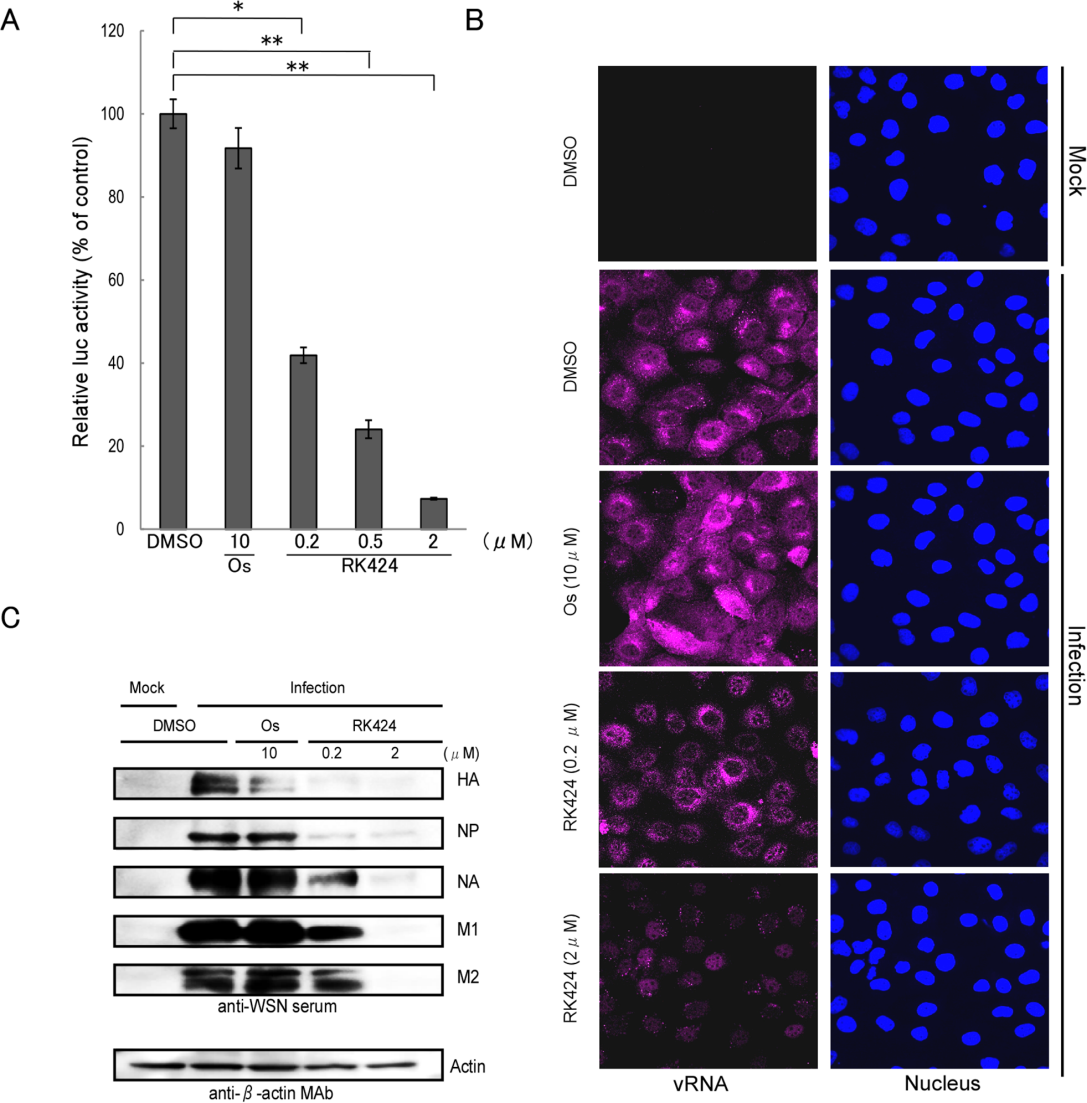
Effect of RK424 on viral transcription, replication, and translation. (A) HEK293T cells were transfected with pCAGGS expression plasmids encoding PB2, PB1, PA, NP, and vNP-luc in the absence or presence of RK424 (0.2, 0.5, and 2 μM). The effect of vRNA transcription was then evaluated by measuring luciferase activity at 48 h post-transfection. Data are expressed as the mean ± SD of three samples in each of three independent experiments. *;p<0.005 and **;p<0.001. (B) MDCK cells were infected with A/WSN/1933 (H1N1) at an MOI of 5 in the absence or presence of RK424 (0.2 and 2 μM) and then fixed at 6 h post-infection. The cells were probed with Quasar 670-labeled probes against the PB2 segment (magenta) and nuclei were stained with Hoechst (blue). Two independent experiments were performed and one representative result is shown. (C) Virus-infected cells were treated with RK424 (0.2 μM and 2 μM) for 18 h and the cell lysates subjected to western blotting with anti-WSN virus serum and an anti-β-actin monoclonal antibody (MAb). Bands representing HA, NP, NA, M1, M2, and actin are indicated. Oseltamivir phosphate (Os) was used as the negative control. Two independent experiments were performed and one representative result is shown.

In contrast to treatment with oseltamivir phosphate (which inhibits the budding step of the viral life cycle), treatment with RK424 led to a reduction in the number, but not the size, of viral plaques (S3 Fig) and it was effective against the oseltamivir resistant virus, A/Tex/131/E119V (H3N2) (Fig 1B) [28]. Furthermore, RK424 did not interfere with viral replication when added to MDCK cells prior to virus inoculation (S4 Fig) and exhibited a prominent antiviral effect at an early stage in the virus life cycle (S5 Fig). These results indicate that the RK424 may target the post-entry and early replication step of the viral life cycle. To examine this further, we first explored the possibility that RK424 affects viral genome replication and/or transcription, both of which occur during the early stages in viral life cycle. To examine the effect of RK424 on vRNP activity (vRNP regulates viral replication and transcription), we performed a mini-genome assay using five expression plasmids harboring influenza A/WSN/1933 (H1N1): PB2/pCAGGS, PB1/pCAGGS, PA/pCAGGS and NP/pCAGGS, which control viral genome replication and transcription, and the luciferase-containing plasmid vNP-luc/pHH21, which encodes a viral-like genome in the absence or presence of RK424. We found that RK424 caused a notable and dose-dependent reduction in luciferase activity (Fig 2A) compared with control (dimethyl sulfoxide (DMSO)) treatment. By contrast, treatment with 10 μM of oseltamivir phosphate did not significantly inhibit luciferase activity.

To confirm the inhibitory effect of RK424 on viral genome replication in infected cells, we next performed single-molecule fluorescence *in situ* hybridization (FISH) analysis with 48 single Quasar 670-labeled DNA oligos against vRNA encoding the PB2 subunit [26, 29] (S3 Table). Large numbers of vRNAs were observed in the cytoplasm of DMSO- and oseltamivir phosphate-treated MDCK cells at 6 h post-infection. By contrast, RK424 treatment resulted in a significant reduction in vRNA expression, and vRNAs mainly localized in the nucleus (Fig 2B).

Furthermore, a reduction in viral protein expression was also detected by western blotting (Fig 2C). Taken together, these results suggest that RK424 affects the function of the vRNP complex (PB2, PB1, PA, and NP), which is associated with viral polymerase activity.

### RK424 inhibits the cytoplasmic localization of NP

**Fig 3:**
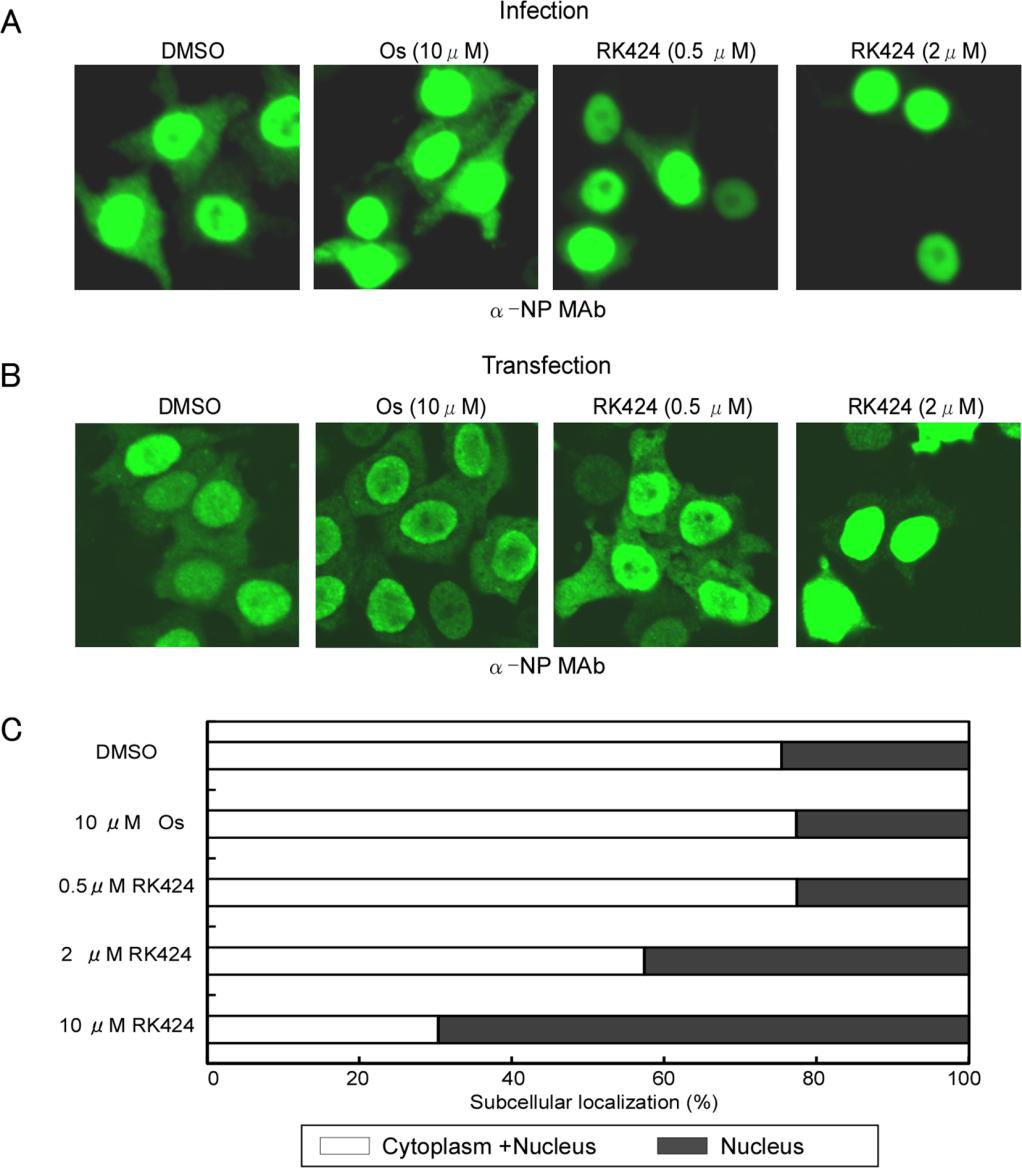
Effect of RK424 on cytoplasmic localization of NP. (A) HeLa cells were infected with A/WSN/1933 (H1N1) virus at an MOI of 10 in the absence or presence of 0.5 and 2 μM of RK424 for 6 h. Subcellular localization of NP was observed by indirect immunofluorescence staining with anti-NP MAb under a confocal laser scanning microscope. Three independent experiments were performed and one representative result is shown. (B) HeLa cells were transfected for 48 h with NP/pCAGGS plasmid in the absence or presence of RK424 (0.5μM and 2 μM). The subcellular localization of NP was observed as described for virus-infected cells. Three independent experiments were performed and one representative result is shown. (C) The percentage of cells showing nuclear localization (black bar) or cytoplasm localization (white bar) of NP was calculated by counting 500 cells per sample. Oseltamivir phosphate (Os) was used as a negative control. Data are expressed as the mean ± SD in each of three independent experiments.

NP is the major component of the vRNP complex [30]. Therefore, to further investigate the effects of RK424 on the nuclear import and export of the vRNP complex in virus-infected cells, we examined NP localization by fluorescence microscopy. We found that NP localized in both the nucleus and cytoplasm at 6 h post-infection in HeLa cells treated with either DMSO (control) or 10 μM of oseltamivir phosphate. By contrast, treatment with RK424 (0.5 or 2 μM) led to nuclear rather than cytoplasmic localization of NP, indicating that the vRNP complex could enter the nucleus but could not be exported to the cytoplasm (Fig 3A). Importantly, the effects of RK424 in virus-infected HeLa cells were also observed in HeLa cells transiently expressing NP (Fig 3B). The cytoplasmic distribution of NP was dependent upon the dose of RK424. As shown in Fig 3C, the cytoplasmic distribution of NP in HeLa cells transiently expressing NP in the absence and presence of 0.5, 2, and 10 μM of RK424 was 75%, 77%, 57%, and 30%, respectively. This shows that RK424 interferes with the nuclear export of NP in the absence of other viral components. Taken together, these results suggest that RK 424 may exert its antiviral effects by inhibiting the function of NP.

### RK424 binds to a small pocket structure within NP that is involved in oligomerization and nuclear export

**Fig 4:**
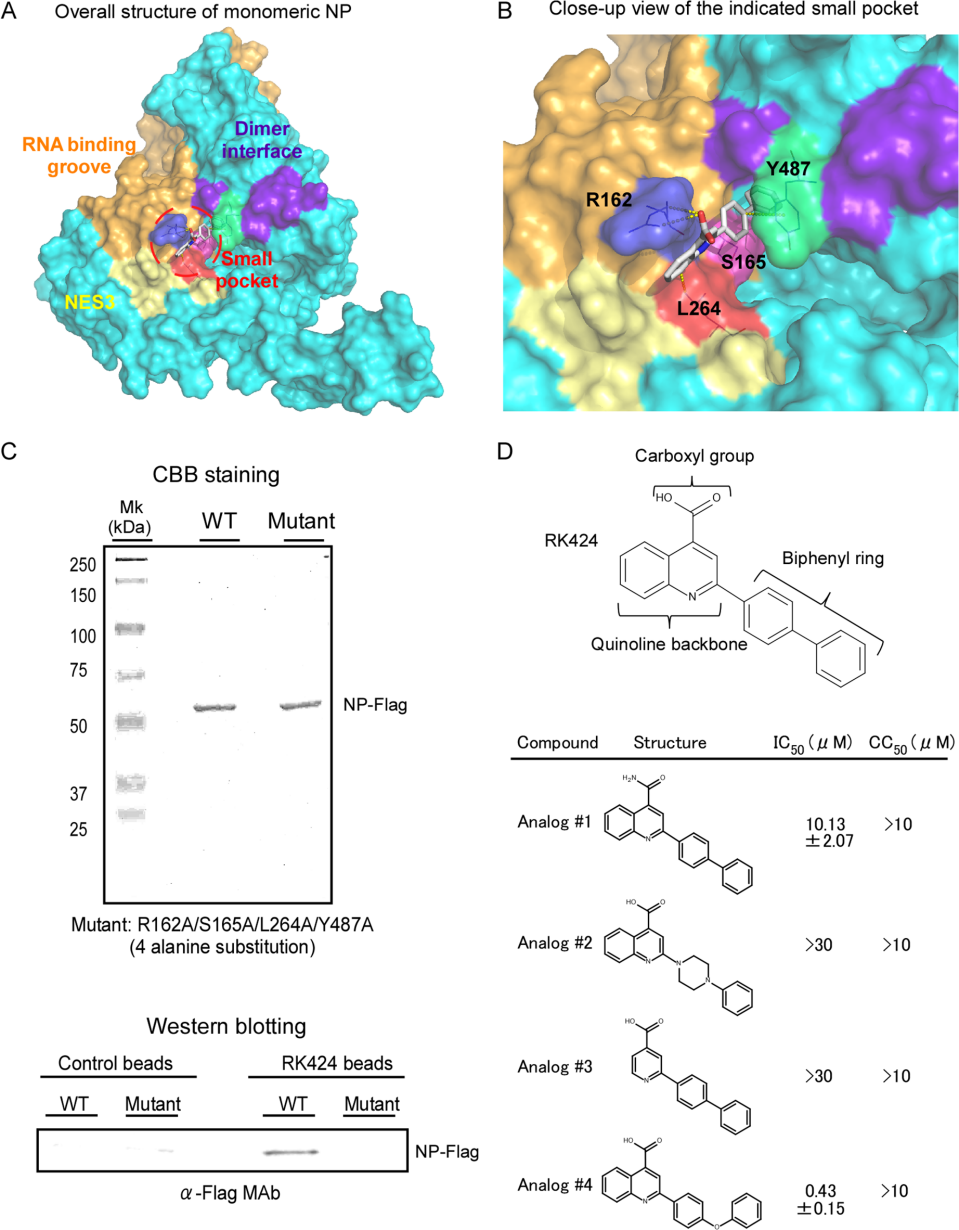
Model for potential binding of RK424 to NP. *In silico* docking analysis was used to predict potential binding sites for RK424 on NP. The configuration with the highest binding energy was visualized using PyMol. (A) Crystal structure of NP. The nuclear export signal (NES) (yellow: amino acid (aa) 256–266), RNA binding grove (orange: aa 1–180), and dimer interface (purple: aa 482–489) are shown on the surface representation. The small pocket is highlighted by red circles. (B) Close-up of the NP small pocket. An electrostatic surface representation of the potential binding site on NP. Amino acids are colored blue (R162), pink (S165), red (L264), and green (Y487). (C) Purified wild-type NP-Flag (WT) and purified mutant NP-Flag (Mut) proteins harboring alanine substitutions at all four potential binding sites (162 165, 264, and 487) were added to RK424 cross-linked affinity beads or uncross-linked beads. Isolated NP-Flag (WT) and mutant NP-Flag (Mut) proteins were run in 10% SDS-PAGE gels and purity was checked by Coomassie Brilliant Blue (CBB) staining (upper panel). The binding of NP to RK424 beads was detected by western blotting with an anti-Flag MAb (lower panel). The positions of the NP-Flag proteins are indicated. Three independent experiments were performed and one representative result is shown. (D) Structure-activity relationship (SAR) analysis of RK424. The *in vitro* antiviral activity (IC_50_) and cell toxicity (CC_50_) of four different structural compounds derived from RK424 were evaluated in a plaque assay and in a WST-1 assay based on MDCK cells. Data are expressed as the mean ± SD of three samples in each of three independent experiments.

To further examine how RK424 inhibits NP function, we used *in silico* docking analysis to create a potential model for the binding of RK424 to NP. To establish unbiased predictive virtual docking models, we obtained the crystal structure of monomeric influenza A/WSN/1933 (H1N1) NP from the Protein Data Bank (PDB) and performed docking studies using AutoDock molecular modeling simulation software [31]. Three potential binding sites were identified; however, the potential binding models showed that the interaction with the highest binding free energy (ΔG) occupied binding site 1 (S6 Fig). Moreover, binding site 1 was surrounded by three functionally important domains: the RNA binding groove (orange) [11], the NP dimer interface (purple), [12] and NES3 (yellow) [18] (Fig 4A and S6 Fig, front side). Therefore, we focused on the binding model based on binding site 1. The interaction map predicted six different configurations for binding site 1, revealing that four amino acid residues (R162, S165, L264, and Y487) were predominantly involved in the interaction with RK424 (S7 Fig); the amino acid residues within binding sites 2 and 3 that were predicted to interact with RK424 did not correlate with any known NP functions (S8 Fig). RK424 occupied a small pocket on NP; configuration 0 had the best fitting score (ΔG of −8.03 kcal/mol) (Fig 4B and S7 Fig).

To confirm the accuracy of this binding model, we first prepared RK424-cross-linked affinity beads (RK424 beads) using a photo-cross-linking method [32] and examined whether RK424 binds to wild-type and mutant NP proteins harboring alanine substitutions at all four potential binding sites (R162A, S165A, L264A, and Y487A). Recombinant wild-type NP (NP-Flag) and Flag-tagged mutant NP proteins (mutant NP-Flag) were purified by FLAG affinity agarose beads. The purity of the purified proteins was checked by running samples in 10% SDS-PAGE gels followed by Coomassie Brilliant Blue (CBB) staining (Fig 4C, upper panel). The purified proteins were then incubated with control and RK424 beads and binding analyzed by western blotting. Wild-type NP-Flag protein bound to RK424 beads but not to control beads; however, the amount of mutant NP-Flag protein bound to RK424 beads was significantly lower than that of wild-type NP-Flag protein (Fig 4C, lower panel). These results suggest that RK424 binds to NP through the amino acid residues predicted by the interaction model.

Therefore, we next explored the structure-activity relationship (SAR) of RK424 using RK424 analogs. Substituting the carboxyl group with an amide group (Analog #1) led to a partial reduction in the antiviral effect, whereas the loss of the biphenyl ring (Analog #2) and quinolone backbone (Analog #3) led to a complete loss of antiviral activity. By contrast, replacing the biphenyl ring with a diphenyl ether moiety (Analog #4) resulted in an antiviral effect comparable with that of RK424 (Fig 4D). Analog #4 showed almost the same antiviral effect (IC_50_ = 0.43±0.15 μM) as RK424 (IC_50_ = 0.48±0.19 μM), but we could not obtain sufficient Analog #4 for further analyses. Analog #4 is synthesized by Wakunaga Pharmaceuticals and is not commercially provided by any supplier. Therefore, we used RK424 for further analysis. Taken together, these results agree with those obtained from the docking model (S7 Fig), i.e., that the carboxyl group, quinoline backbone, and biphenyl ring of RK424 may be required for the inhibitory effects of RK424.

### RK424 disrupts NP-RNA and NP-NP interactions, thereby inhibiting NP oligomerization

**Fig 5:**
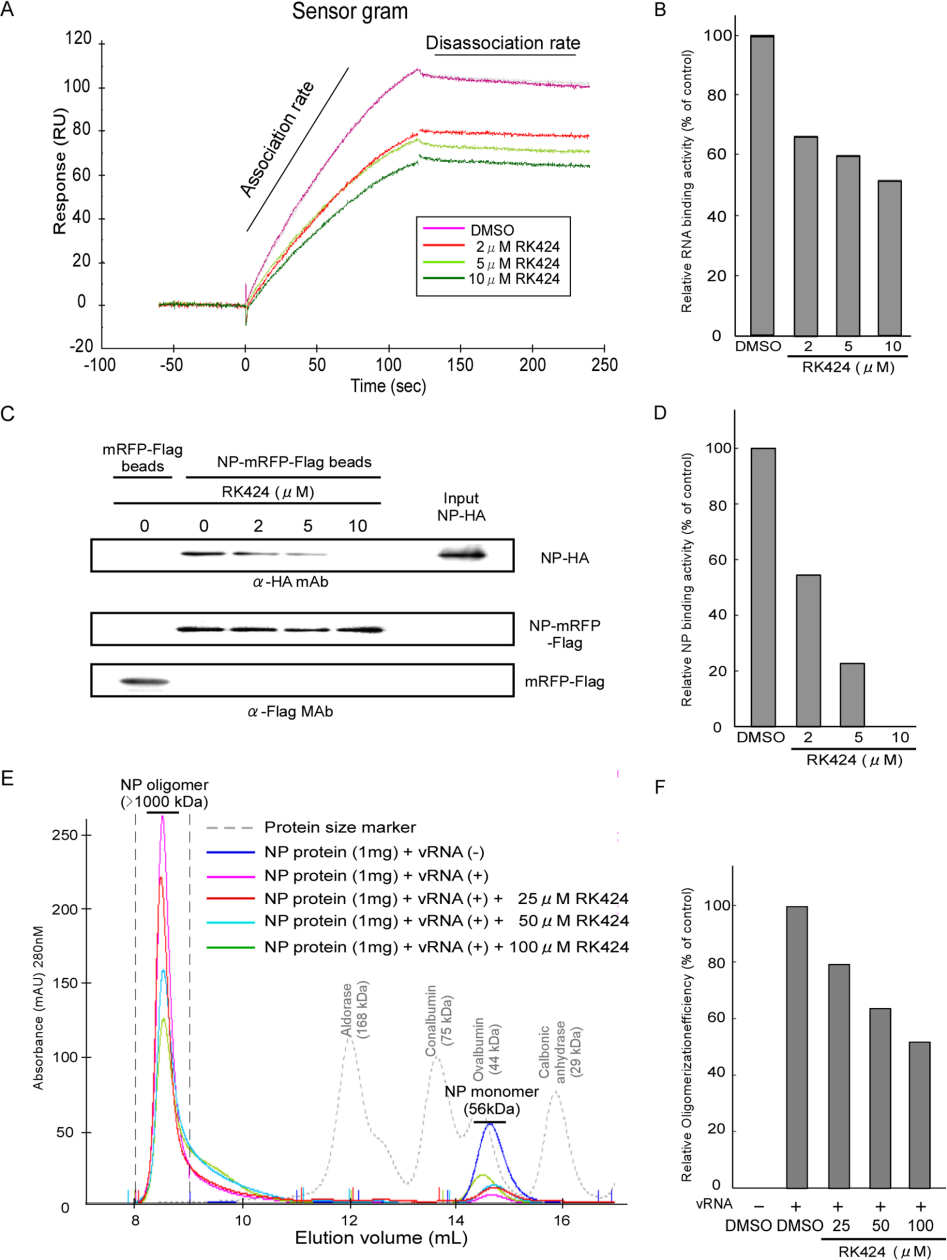
Effect of RK424 on the NP-RNA and NP-NP interactions, and on NP oligomerization. (A) Sensorgram showing the interaction between NP and RNA in the absence or presence of RK424. Two independent experiments were performed and one representative result is shown. (B) Quantification of RNA binding activity. (C) Purified NP-HA proteins were incubated with RK424 for 1 h followed by incubation with FLAG agarose beads coupled to mRFP-Flag (mRFP-Flag beads) or NP-mRFP-Flag proteins (NP-mRFP-Flag beads). Binding of NP-HA to mRFP-Flag or NP-mRFP-Flag beads and the input NP-HA were detected by western blotting with an anti-HA MAb. The amount of protein bound to FLAG agarose beads was detected with an anti-Flag MAb (loading control). Three independent experiments were performed and one representative result is shown. (D) Pull-down assay to examine NP binding activity. (E) Purified NP proteins were pre-incubated with RK424 followed by incubation with vRNA synthesized by *in vitro* transcription. The incubated samples were injected onto a Superdex 200 Increase size exclusion column. The integrated peaks between and elution volume of 8 mL and 9 mL were evaluated to measure the oligomerization rate of NP. Two independent experiments were performed and one representative result is shown. (F) Oligomerization rate of NP as assessed by size exclusion chromatography.

The four amino acids residues that play a potential role in RK424 binding to NP reside within different functional domains. These domains are involved in (i) oligomerization of NP via NP-RNA and NP-NP interactions, and (ii) nuclear export of NP. We first used surface plasmon resonance (SPR; Biacore) to examine the effect of inhibiting the NP-RNA interaction. A 24-mer RNA oligomer was biotinylated at the 5’-end (to facilitate immobilization) and methylated at the 2’-OH of ribose (to provide nuclease resistance) was immobilized on an SA sensor chip. We chose a 24-mer because studies show that this length approximates the binding distance between NP and vRNA [33,34,35,36]. Samples containing RK424 (10 μM) showed a reduced binding response to RNA. This reduction was dependent upon the dose of RK424. The RNA binding observed for treated samples was almost 50% less than that observed for control samples (Fig 5A and 5B). Notably, RK424 affected the rate of association without affecting the rate of disassociation. This suggests that RK424 interferes with the binding of NP to RNA.

We next used a pull-down assay incorporating NP proteins fused to different tags to examine whether RK424 caused the NP-NP homo-oligomer to dissociate. In the absence of RK424, a purified recombinant wild-type NP protein fused to a HA tag (NP-HA) bound to FLAG agarose beads coupled to purified recombinant wild-type NP protein fused to mRFP-Flag-tag (NP-mRFP-Flag beads); however, NP-HA did not bind to control FLAG agarose beads coupled to mRFP-Flag protein (mRFP-Flag beads). RK424 inhibited the binding of NP-HA to NP-mRFP-Flag beads in a dose-dependent manner (Fig 5C and 5D). These results suggest that RK424 inhibits the NP-NP interaction.

NP shapes the main backbone and regulates the conformation of vRNP by interacting with RNA and with itself (NP-NP homodimers) [11,34,37]. Therefore, to examine whether RK424 prevents the formation of large NP oligomer/vRNA complexes, we incubated purified NP proteins (S9A Fig) with vRNA encoding the M segment (synthesized by *in vitro* transcription; S9B Fig) in the absence or presence of RK424. Next, the incubated samples were subjected to size exclusion chromatography and the particle sizes monitored using an ÄKTA purifier and UNICORN software. In the absence of RNA, purified NP proteins exist in equilibrium as monomers and trimers/tetramers (S9C Fig upper panel); this equilibrium is regulated by the salt concentration [33]. Salt concentrations higher than those encountered under physiological conditions shift the equilibrium toward trimers/tetramers, whereas lower salt concentrations shift it toward monomers. In solution, the NP trimers/tetramers gradually dissociate to form monomers [12]. Thus, we maintained the purified NP proteins in a 100 mM NaCl solution at 4°C for 48 h. This shifted the equilibrium toward monomer formation, a situation confirmed by size exclusion chromatography (S9C Fig lower panel). The elution peak for NP proteins incubated in the absence of vRNA occurred at around 14.6 mL, and was attributed to NP monomers; however, molecular mass of the NP protein eluted in this peak was estimated to be around 40 kDa, which is smaller than the molecular mass measured by 10% SDS-PAGE (56 kDa) (Fig 5E and S9A Fig) [11]. This suggests that the NP molecule adopts a more globular and compact shape than the proteins used for calibration. On the other hand, NP proteins incubated in the presence of vRNA but in the absence of RK424 eluted at around 8.5 mL; this peak was attributed to the presence of large NP oligomers (as calculated from the standard curve) (Fig 5E). These results show that the NP protein was able to form large oligomers by interacting with vRNA. Next, we examined the inhibitory effect of RK424 on NP oligomer formation. In the presence of vRNA and RK424, the peak height of the large NP oligomer was reduced; instead, a broad peak was observed and the elution volume increased to around 10 mL (Fig 5E). This suggests that RK424 inhibited NP-NP and NP-RNA interactions, thereby interfering with oligomerization. Because the NP peak in the presence of vRNA occurred between 8 mL and 9 mL in the absence of RK424, we quantified the rate of NP oligomerization by evaluating the peak integration between these elution volumes. There was a dose-dependent reduction in the rate of oligomerization in the presence of RK424 (Fig 5F). Taken together, these results demonstrate that RK424 inhibits NP oligomerization by disrupting NP-RNA and NP-NP interactions.

### RK424 inhibits the nuclear export of NP and NP-CRM

**Fig 6:**
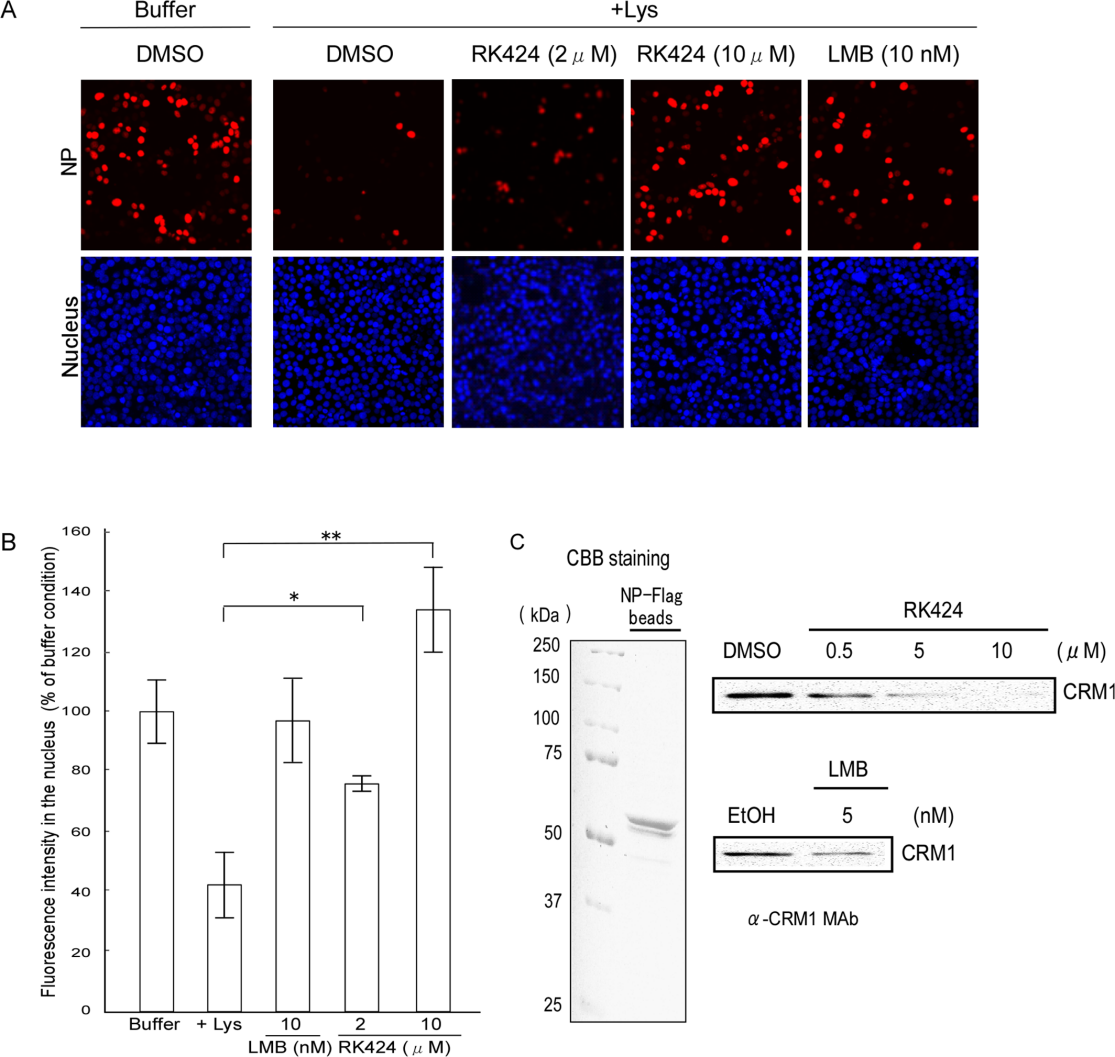
Effect of RK424 on nuclear export of NP and the binding of NP to CRM1. (A) *In vitro* nuclear export assay incorporating NP-mRFP-Flag. Cells expressing NP-mRFP-Flag were permeabilized with digitonin and incubated for 1 h without (Buffer) or with HeLa cell lysate (+Lys) in the presence of the RK424 at the indicated concentrations (upper panels). Leptomycin B (LMB) was used as a positive control (LMB inhibits nuclear export of NP). Nuclei were stained with Hoechst 33342 (lower panels). The export of NP-mRFP-Flag was monitored by measuring mRFP fluorescence in the nucleus. Three independent experiments were performed and one representative result is shown. (B) Quantification of mRFP fluorescence in the nucleus. The nuclear localization of NP-mRFP-Flag was determined by monitoring mRFP fluorescence in the nucleus (> 100 cells). Data are expressed as the mean ± SD of three independent experiments. *;p<0.05 and **;p<0.005. (C) RK424 inhibits the binding of NP-mRFP-Flag to CRM1. FLAG agarose beads coupled to NP-Flag (NP-flag beads) were subjected to SDS-PAGE and the purity of the NP-Flag protein was checked by Coomassie Brilliant Blue (CBB) staining (left panel). HeLa cell lysate was incubated with NP-Flag beads in the absence or presence of RK424 (0.5, 5, or 10 μM). LMB was used as a positive control. Binding of CRM1 to NP was detected by western blotting with an anti-CRM1 MAb (right panel). EtOH and DMSO were used as vehicle controls. The positions of NP-Flag and CRM1 are indicated. Two independent experiments were performed and one representative result is shown.

In addition to the NP amino acids involved in NP-RNA and NP-NP interactions, the docking model showed that RK424 partially bound to amino acids that form the NES3 domain (Fig 4B). To investigate the inhibitory effect of RK424 on the nuclear export of NP, we performed an *in vitro* nuclear export assay using digitonin-permeabilized, semi-intact HeLa cells (Fig 6A and 6B). The nuclear export activity of NP-mRFP-Flag protein was examined by observation and quantification of mRFP fluorescence under a confocal laser scanning microscope. In the absence of cell lysate, many cells showed mRFP fluorescence (Fig 6A; Buffer). By contrast, the number of cells expressing NP-mRFP-Flag was clearly lower in presence of cell lysate (Fig 6A; +Lys). The fluorescence intensity of these cells was reduced to 42% (of that in untreated cells) in the presence of cell lysate (Fig 6B). In the presence of leptomycin B (LMB), many cells showed levels of mRFP fluorescence comparable with those in buffer-treated samples and 97% of the fluorescence intensity was retained in the nucleus compared with that in buffer-treated cells (Fig 6A; LMB, and Fig 6B). The fluorescence intensity in the presence of 2 μM and 10 μM RK424 was 76% and 134% of that in buffer-treated cells, respectively (Fig 6A; RK424, and Fig 6B), indicating that RK424 inhibited the nuclear export activity of NP.

Next, to investigate whether RK424 inhibits nuclear export by preventing NP from binding to CRM1, we examined the binding of endogenous CRM1 to NP in the presence or absence of RK424. CRM1 bound to FLAG agarose beads coupled to the purified NP-Flag protein (NP-Flag beads); however, binding was inhibited in the presence of RK424 (5 μM or 10 μM) and LMB (positive control) (Fig 6C). RK424 inhibited CRM1 binding to NP-Flag beads in a dose-dependent manner.

### The small pocket within NP has a unique surface structure

When developing new drugs, identifying potential off-target effects is very important. With this in mind, we superimposed the NP pocket on to other known binding pocket surfaces to identify structural similarities. To do this, we searched two comprehensive databases: Pocket Similarity Search using Multiple-sketches (PoSSuM) [38] and ProBiS [39]. PoSSuM covers 1.8 million known and potential binding sites in the PDB, and ProBiS covers over 29,000 non-redundant proteins in the PDB. These databases allow researchers to compare the protein structure of the test molecule with those in the data base. In addition to the NP Pocket Similarity Search, we also examined the influenza A PA pocket, which is positioned at the active site of the endonuclease domain because this structure is a potential novel antiviral target [40,41,42] (S10A Fig). The ProBiS search identified 20 pocket structures that were similar to the PA structure, some of which are involved in host cell function (S10B and S10C Fig). By contrast, no proteins with a structure similar to the NP pocket were identified, suggesting that the NP pocket has a unique surface structure. Thus, targeting the NP structure rather than the PA structure may be much less likely to cause off-target effects.

### The amino acid residues that form the predicted RK424 binding pocket within NP are highly conserved and play essential roles in viral replication

**Fig 7:**
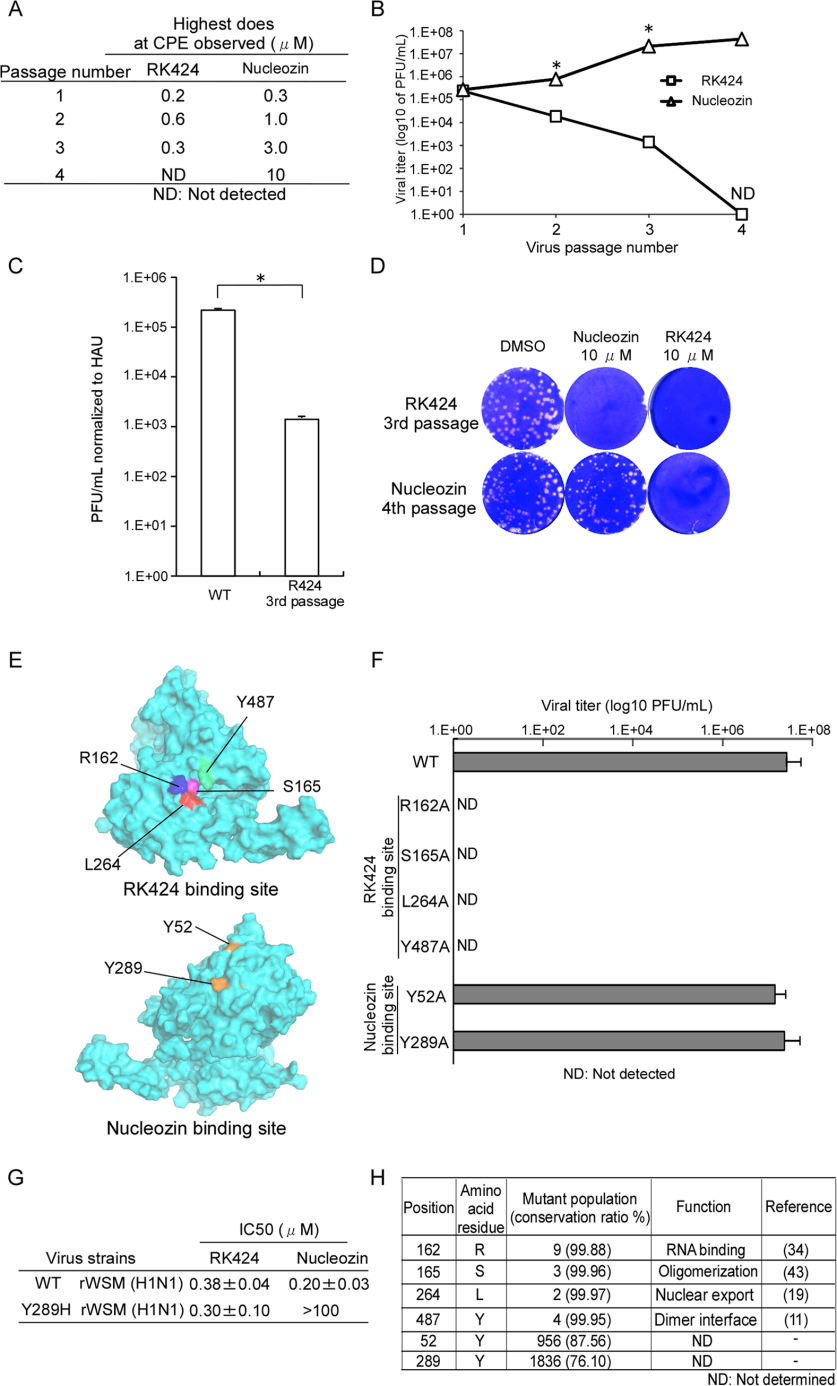
Production of mutant viruses and conservation of amino acids at RK424 and nucleozin binding sites. (A) *In vitro* selection of escape viruses in the presence of serial dilutions of RK424 and nucleozin. RK424 concentration range: 1^st^; 0.1–2 μM, 2^nd^; 0.2–3 μM, 3^rd^; 0.3–3μM and 4^th^; 0.4–3 μM. Nucleozin concentration range: 1^st^; 0.1–2 uM, 2^nd^; 0.3–3 μM, 3^rd^; 1–5μM and 4^th^; 3–10 μM. The highest concentrations of compound that elicited CPE at each passage are listed. (B) Plaque titration of escape viruses in each passage. Values represent the mean ± SD of three independent experiments. The symbol (*) indicates statistically significant differences in mean viral titer (PFU/mL) between RK424 passaged virus and nucleozin passaged virus at each passage number.*;p<0.001. (C) Viral titer of RK424 3^rd^ passaged virus and nucleozin 4^th^ passaged virus stocks normalized to absolute virion numbers (based on hemagglutination units; HAU). Values represent the mean ± SD of three independent experiments. *;p<0.005. (D) Sensitivity of RK424- and nucleozin-selected viruses to DMSO (left well), 10 μM nucleozin (middle well) and 10 μM RK424 (right well). Two independent experiments were performed and one representative result is shown. (E) Models showing the binding sites on NP targeted by RK424 and nucleozin. (F) Production of recombinant viruses harboring R162A, S165A, L264A, Y487A, Y52H, and Y289H mutations in NP. HEK293T/MDCK cells were transfected with plasmids expressing four viral proteins (PB2, PB1, PA, and NP) and eight vRNAs (PB2, PB1, PA, HA, NP, NA, M, and NS). Plasmids expressing mutant NP proteins and genomes were used as a substitute for the WT plasmid when generating the R162A, S165A, L264A, Y487A, Y52H, and Y289H NP mutant viruses. After 72 h of transfection, supernatants were harvested and used in plaque assays on MDCK cells. Data are expressed as the mean ± SD of three independent experiments. (G) The 50% inhibitory concentration (IC_50_) of RK424 and nucleozin against rWSN and Y289H rWSN viruses were evaluated in a plaque assay in the absence or presence of RK424 and nucleozin (0–100 μM). Data are expressed as the mean + SD of three samples in each of three independent experiments. (H) Conservation of RK424 binding sites (R162, S165, L264, and Y487) and nucleozin binding sites (Y52 and Y289) in human, avian, and swine influenza A viruses. Perl script was used to analyze 7683 NP sequences derived from human, avian, and swine influenza A viruses.

Finally, to demonstrate the accuracy of our docking model and confirm that RK424 binds to the binding pocket within NP, we selected escape mutant viruses by passaging an IAV in the presence of RK424. In this experiment, we selected nucleozin as the positive control, because nucleozin exerts a potent antiviral effect via its ability to cross-link two NP molecules [25]. The structures of the NP inhibitors are listed in S11 Fig. The selection was carried out for a total of four passages with serially increasing concentrations of RK424 and nucleozin. In the cells passaged with RK424, CPE was observed until 3^rd^ passage, but no CPE occurred at the 4^th^ passage. On the other hand, as the number of passages increased, the concentration of nuclozin at which CPE occurred was up to 10 μM by the 4^th^ passage (Fig 7A). Furthermore, viral replication imposed by nucleozin treatment increased as the number of passages increased but viral replication imposed by RK424 treatment decreased (Fig 7B).These results suggest that RK424 makes viral infected cells produce more propagation-deficient virions than fully infectious virions. To confirm this notion, we evaluated the infectivity of RK424 3^rd^ passage virus by comparing the viral titer of this virus normalized to absolute virion numbers (estimated by hemagglutination units; HAU) with that of the initial WT viral titer used in the selection of the escape mutant. The titer of the RK424 3^rd^ passage virus was 1.41×10^3^ PFU/mL per HAU, which was lower than that of the WT (2.19×10^5^ PFU/mL per HAU) (Fig 7C). These results indicate that the number of propagation-deficient virions produced by RK424 3^rd^ passage virus had increased. After four passages, we confirmed sensitivity of RK424- and nucleozin-selected viruses to 10 μM RK424 and nucleozin (Fig 7D). Nucloezin 4^th^ passage virus exhibited resistance to 10 μM nucleozin but not RK424. However, RK424 3^rd^ passage virus was susceptible to both 10 μM RK424 and nucleozin. These results showed that nucleozin-resistant viruses appeared after four passages with nucleozin but RK424 3^rd^ passage viruses were unable to acquire resistance to RK424. Sequence analysis revealed that nucleozin 4^th^ passage virus had three amino acid mutations (Y52H, Y289H and Y313S) which were previously reported to be result in resistance to nucleozin [25], but no amino acid mutation was detected in RK424 3^rd^ passage virus, which also support the result of sensitivity of RK424- and nucleozin-selected viruses to RK424 and nucleozin.

Therefore, we next used a reverse genetics approach to generate mutant viruses harboring an alanine substitution at each potential RK424 binding site within NP (Fig 7E). We successfully generated recombinant wild-type WSN (rWSN) and mutant rWSN viruses harboring Y52H and Y289H mutations within the NP, which have previously been shown to be major mutations resulting in resistance to nucleozin [24,25]. Although these viruses grew well, none of the mutant rWSN viruses harboring alanine substitutions at potential RK424 binding sites were rescued at 72 h after transfection (Fig 7F). Interestingly, we found that although nucleozin did not inhibit the replication of Y289H rWSN, RK424 did (Fig 7G). These results suggest that the binding of RK424 to the amino acids in the NP pocket (or to a site different from that recognized by nucleozin) may inhibit a process that is essential for the viral life cycle. In addition, we investigated the conservation of amino acid residues within the potential RK424 binding site (R162, S165, L264, and Y487), and within the nucleozin binding site (Y52 and Y289), among IAVs of human, avian, and swine origin (Fig 7H). To do this, we used perl script to analyze NP sequences obtained from the NCBI Influenza Virus Resource. The predicted amino acids responsible for RK424 binding showed > 99% conservation among avian, human, and swine IAVs; however, the tyrosine (Y) residues at positions 52 and 289 (which are crucial for nucleozin binding) were poorly conserved. These data indicate that mutations at potential RK424 binding residues are functionally restricted. In actuality, these amino acid residues are reported to regulate various NP functions during the viral life cycle [11, 19, 34, 43]. These findings support the hypothesis that the small pocket within NP may be a promising target for antiviral drugs, particularly a multifunctional NP inhibitor.

## Discussion

Here, we identified a novel small pocket structure within NP as a potential target for antiviral drugs. Moreover, the *in vivo* co-administration (RK424 and oseltamivir phosphate) data and the effectiveness of RK424 against the oseltamivir resistant virus indicated that RK424 inhibited viral replication via a pathway different that of existing antiviral drugs (neuraminidase inhibitors). Further analyses showed that RK424 bound to the small pocket of NP and interfered with three core NP functions: i) the NP-RNA interaction, ii) the NP-NP interaction, and iii) the nuclear export of NP. In addition, we demonstrated that the NP pocket structure comprised three functional domains: the RNA binding groove, the dimer interface, and NES3. Previous reports [11, 12, 34, 43] show that several amino acid residues within the RNA binding groove and dimer interface are essential for NP-RNA and NP-NP interactions. Moreover, Chutiwitoonchai *et al*. showed that NP-NES3 is the most important NP-NES sequence for nuclear export of NP and viral replication [19]. Taken together, these reports strongly suggest that these three domains regulate the oligomerization and nuclear export of NP, thereby maintaining the structure and function of the vRNP, which plays a key role in viral replication. Moreover, we showed that the identified NP pocket has a surface structure different from that of host cell molecules, suggesting that it may be an ideal drug target. Finally, we generated predicted configurations for a potential RK424 binding site (binding site 1) and showed that RK424 partially overlapped all three functional domains and interacted predominantly with four amino acids residues, R162, S165, L264, and Y487, which play important roles within the three functional domains [11,18,34,43]. According to these binding models, RK424 inhibits the oligomerization and nuclear export of NP, suggesting that the NP pocket is involved in multiple NP functions.

*In silico* docking studies incorporating RK424 and monomeric NP identified three potential binding sites for RK424 (S6 Fig). Binding site 1 is surrounded by the three different functional domains (S7 Fig), whereas potential binding sites 2 and 3 are not (S8 Fig). The docking model for binding site 2 revealed that RK424 interacts with four amino acids residues: R422, S450, R452, and D455 (S8 Fig). However, these amino acid residues are not well-conserved among influenza virus strains and no relationship between these residues and viral protein function has been reported. The model for potential binding site 3 revealed that RK424 binds to only three residues, R461, G462, and P474; this binding model had the lowest binding energy of all models tested (S8 Fig). Although these three amino acids are well-conserved between species, their functional importance for viral replication is unclear. However, a previous report used mutational analysis and plasmid-driven reverse genetics to show that P474 has no significant role in viral replication [44]. Taken together, these data suggest RK424 is unlikely to bind to sites 2 and 3. By contrast, the predicted NP pocket at potential binding site 1 comprises functionally well-established domains, and the configuration of RK424 at this pocket has high binding energy. The experiments involving RK424 cross-linked beads support the data from the docking model. Moreover, amino acid residues R162, S165, L264, and Y487 are well-conserved and play roles in essential viral protein function. The data derived from these docking models may explain why RK424 shows a broad spectrum of activity against IAV.

The results of the SAR experiments showed that the carboxyl group, the quinoline backbone, and the biphenyl ring of RK424 are essential for its antiviral effects. Fitting these results to configuration 0 (the RK424 binding model) revealed that the NP pocket had the best fitting score. The carboxyl group of RK424 forms a hydrogen bond with R162 within the NP pocket and the phenyl ring derived from the quinolone backbone of RK424 interacts with the L264 side chain via Van der Waals forces. Furthermore, the biphenyl ring of RK424 forms a CH-π hydrogen bond with the Y487 side chain. The S165 side chain does not bind directly to RK424 in this model; however, other configuration models (configuration 1, 2, 3, and 5) suggested a direct interaction between residue S165 and the carboxyl group or the phenyl ring derived from the quinolone backbone of RK424. Moreover, other amino acid sequences, such as F488 and F489, were also predicted to interact with RK424 (S7 Fig). Thus, it is difficult to predict the exact mechanism via which RK424 binds to the NP pocket from this study. Although preliminary data from the potential docking models allow us to speculate as to how RK424 binds to the NP pocket, further studies (e.g., co-crystallization of the NP protein/RK424 complex) are needed to definitively identify the precise interaction model.

The vRNP, comprising the viral genome, polymerase subunits PB2, PB1, and PA, and NP, regulates viral transcription and replication in the host cell nucleus, and is maintained via NP-NP and NP-RNA interactions [11,34,37]. After viral transcription and replication, the vRNP components must be exported from the nucleus to the cytoplasm, where they assemble at the cell membrane prior to budding. This process is crucial for the viral life cycle; therefore, it is a potential target for novel antiviral agents. RK424 inhibited several important processes; namely, the RNA-induced oligomerization and nuclear export of NP. Three of the amino acid residues predicted to be involved in RK424 binding (R162, S165, and Y487) are located within domains involved in NP oligomerization. Residues R162 and S165 are located in the RNA binding groove of NP; however, they play different functional roles. Many positively-charged residues, e.g., arginine (R) and lysine (K), are present in the RNA binding groove, where they interact with negatively-charged RNA molecules. In addition, the electropositive groove between the head and the body domains of NP (NP-G2) contains four arginine residues, R150, R152, R156, and R162 [34]. Mutating these arginine residues to alanine leads to a significant reduction in the affinity of NP for RNA, suggesting that R162 is involved in RNA binding [34]. On the other hand, a previous report showed that the phosphorylation status of residue S165 within NP is closely associated with NP oligomerization. An S165D mutant (which mimics S165 phosphorylation) attenuates the NP-NP and NP-RNA interactions by stabilizing the monomeric structure [43]. In addition, the S165A mutation attenuates vRNA transcription, thereby preventing the recovery of recombinant virus [45]. Moreover, the crystal structure of an ∆402–429 NP mutant suggests a new NP-NP interaction model that does not involve the tail loop binding pocket [12]. This interaction comprised amino acid residues 149–167 and 482–498 of NP. Notably, mutating Y487 attenuates the transcription and replication of vRNA by disrupting the NP-NP interaction [12]. RK424 inhibited NP oligomerization by directly inhibiting the NP-RNA and NP-NP interactions, thereby causing a significant reduction in viral RNA synthesis. This suggests that RK424 may disrupt NP functions that are dependent on residues R162, S165, and Y487. After NP oligomerization, vRNP is exported from the nucleus to cytoplasm via the NS2/NEP and M1 complex pathways [13,14,15,16,17]. Recent studies suggest that nuclear export of NP is closely associated with vRNP export because NP, which has an NES, is a major component of the vRNP [18,20]. NP contains three functional NESs: amino acids 24–49 (NES1), 183–197 (NES2), and 248–274 (NES3) [18]. Export via NES3 is CRM1-dependent whereas export through NES1 and NES2 is CRM-independent [18]. Our previous study indicated that mutating NP-NES1 and NP-NES2 did not alter their cytoplasmic localization, and had only a partial effect on replication kinetics; however, nuclear export of an NES3 mutant was attenuated and mutant virus could not be rescued by reverse genetics [19]. Two hydrophobic residues in particular, L264 and L266, play an essential role in nuclear export, CRM-1 binding, and viral production [19]. The present study showed that LMB (a specific inhibitor of CRM1) inhibited the cytoplasmic localization and nuclear export of NP. These results also suggest that nuclear export of NP is mostly CRM1-dependent. A nuclear export assay using digitonin-permeabilized cells showed that NP was able to move from the nucleus to the cytoplasm by interacting with CRM1, and that RK424 inhibited this process. Interestingly, the present study shows that RK424 directly binds to NP and inhibits its binding to CRM1, whereas LMB covalently binds to S528 within CRM1, thereby inhibiting NP nuclear export by disrupting CRM1 binding to the NES [46]. The docking model described herein showed that RK424 partially obstructs NP NES3 by binding to L264, which is critical for nuclear export. Based on this model, binding of RK424 to the NP pocket may inhibit its nuclear export by interfering with CRM1 binding. Furthermore, nuclear export of NP is closely associated with NP oligomerization [14]. The NES, which comprises many hydrophobic amino acid residues, is presented at the surface of the oligomeric NP [18], and the hydrophobicity of NP increases upon oligomerization; therefore, surface exposure of the NES may be associated with NP oligomerization [47]. Indeed, the alanine substitution mutants, E339A and R416A, completely abrogate NP oligomerization, thereby preventing it from localizing to the cytoplasm [11,18,48]. Thus, by inhibiting NP oligomerization, RK424 may also inhibit the nuclear export of NP.

Predicting possible side effects is an important issue when designing new drugs. The potential utility of a molecule as a targetable drug can be examined by comparing the structure of its target binding pocket with the structures of host proteins [49]. Molecular targets that show high similarity to host molecules may cause severe medical problems; such molecules should not be pursued. The NP pocket structure identified herein showed no similarity to any other molecules in the database. By contrast, the pocket in influenza A PA which regulates endonuclease activity and is a promising target for the development of new anti-influenza drugs [40,41,42], showed high similarity to several host molecules. This suggests that the structure of the NP pocket is unique, making it a more promising target for antiviral target drugs. Drugs that target this binding pocket would be expected to have fewer side effects.

Recently, several compounds targeting NP have been identified as novel antiviral drug candidates, including NLS-binding compounds (e.g., mycalamide analogs), NP salt bridge inhibitors (e.g., compound 3), and NP-aggregation inducers (e.g., nucleozin) [22,23,24,25]. NLS-binding compounds and NP salt bridge inhibitors have weak antiviral effects (sub-micromolar order) because they disrupt only one of the NP functions. On the other hand, nucleozin inhibits multiple NP functions and has a potent antiviral effect (nanomolar order). These findings suggest that developing a drug that inhibits multiple NP functions will be extremely effective because NP is the most abundant multifunctional viral protein in infected cells [50]. However, viruses rapidly develop resistance to nucleozin because its target amino acids (Y52 and Y289) do not play an essential role in the viral lifecycle [24,25]. Furthermore, these residues are not highly conserved among IAVs. In particular, swine influenza viruses harbor a natural Y289H mutation, making them resistant to nucleozin [24]. This is the reason for the rapid appearance of resistant viruses and the limited antiviral spectrum of nucleozin. RK424 inhibited several NP functions and showed potent antiviral effects *in vitro* against many different subtypes of IAVs including currently circulating A/California/7/2009 (H1N1) virus and the highly pathogenic avian influenza virus strains, A/Ck/Yamaguchi/7/2004 (H5N1) and A/Anhui/1/2013 (H7N9). These results support the findings that RK424 targets conserved amino acids in different virus strains. Indeed, although we were able to generate nucleozin-resistant virus, we were unable to generate the RK424-resistant virus, even after four passages (Fig 7A–7D). The inability to generate resistant virus, coupled with the inability to rescue viruses harboring R162A, S165A, L264A, and Y487A mutations indicates that RK424 inhibits processes that are essential for the viral life cycle. For example, RK424 inhibits NP-RNA binding and the nuclear export of NP. These processes are important for viral genome packaging into virions. Thus, RK424 might prevent viral genome packaging, resulting in the production of propagation-deficient virions lacking viral genome RNA. These observations may also provide reasons why a RK424 resistant virus was not generated. However, further experiments are needed to fully characterize the underlying mechanism(s).

In addition to demonstrating antiviral activity *in vitro*, we also showed that RK424 exhibited antiviral effects *in vivo*. Remarkably, significant (~2 log) reductions in viral titers in the lung were observed after day 6 post-infection. However, mice body weight was lower after the administration of 10 mg/kg RK424 than after the administration of 10 mg/kg oseltamivir phosphate, which resulted in no weight loss, and recovery from morbidity was modest in the 10 mg/kg RK424-treated group. Therefore, viral titers may rebound after day 6 post-infection and thus mice may continue to succumb to viral infection. Another group has reported that administration of 50 mg/kg T-705 for 5 days, beginning 1 h post-infection, results in significantly lower(~2 log) viral titers (3.50 log_10_ PFU/mL) in the lung than in negative control group (5.47 log_10_ PFU/mL). This was observed after day 6 post-infection, but recovery from morbidity was only 20% of that of the negative control group [51]. These results also suggest that ~2 log reductions in viral titers in the lung after cessation of antiviral compound administration are not enough to prevent viral replication and a rebound in viral titers, which can cause severe morbidity *in vivo*. The reason for this limited antiviral effect of RK424 *in vivo* may be because RK424 is highly lipophilic, a property that affects important parameters such as its solubility, absorbency, and stability, all of which affect PK [52]. Thus, the free plasma concentration of RK424 is not sufficient to exert a potent antiviral efficacy *in vivo*. RK424 has a high plasma protein binding rate because of its high lipophilicity. The efficacy of a drug is affected by the degree to which it exists in a form free from plasma proteins (free drug) within blood plasma. Unbound drugs can traverse cell membranes and become absorbed by target tissues, where they can interact with the drug target and exhibit pharmacologic effects. The plasma protein binding of RK424 is 99.6%, which means only 0.4% of the drug exists in free form in blood plasma. The C_max_ of RK424 (39200 ng/mL) is more than 200 times higher than the IC_50_ concentration used *in vitro* (0.5 μM = 162.7 ng/mL), but the free drug maximum concentration (C_free max_) was 156.8 ng/mL because almost all of the drug was bound to plasma proteins. Taking into account this property, the free plasma exposure of RK424 was adjusted to obtain the *in vitro* IC_50_ (0.5 μM = 162.7 ng/mL) concentration by the intraperitoneal administration of 10 mg/kg RK424 twice per day, but the antiviral effect was limited. This may mean that it will be necessary to increase the plasma free drug concentration to arrive at the *in vitro* IC_90_ concentration (1.2 μM = 390.5 ng/mL). However, the intraperitoneal administration of RK424 at 10 mg/kg did not result in the *in vitro* IC_90_ concentration. We could not increase the dose of RK424 further, because RK424 precipitated from the viecle solution. The oral administration route provides a way to administer a drug with low solubility to animals. However, to achieve and maintain the *in vitro* IC_90_ concentration (1.2 μM = 390.5 ng/mL) in plasma via oral administration, multiple does of around 100 mg/kg RK424 would be needed because the bioavailability and t_1/2_ of oral administration are lower than those obtainable by intraperitoneal administration. Indeed, to design a multiple dosing regimen precisely, the PK study has to be done as a multiple dose of RK424. Moreover, the Vd (583mL/kg) parameter, which is lower than mouse total body water (725 mL/kg), suggests that RK424 may not be efficiently absorbed into target tissues. In conclusion, the PK properties of RK424 are insufficient to exert an antiviral effect *in vivo*. To acquire antiviral activity *in vivo*, the pharmacologic parameters of RK424 will need to be optimized by introducing changes in its chemical structure.

To summarize, the results presented herein highlight a novel strategy for developing new drugs that target the NP pocket; such drugs may have multifunctional effects and reduce the emergence of drug-resistant viruses. One possible approach to developing a potent antiviral drug targeting the NP pocket is to optimize the structure of RK424. This could be achieved by replacing its aromatic rings, the cause of its high lipophilicity, with aromatic heterocyles or by introducing side chains modified with hydrophilic groups, as long as these changes do not affect its antiviral activity. In addition to this approach, target-oriented drug screening may be a more desirable approach because the NP pocket has a very unique surface structure and the amino acid residues within this pocket are highly conserved. These characteristics make the NP pocket an attractive target for drug development. Thus, another promising strategy is computational virtual screening, which is a useful way of identifying small molecular compounds with backbone structures that differ from those of RK424.

## Materials and Methods

### Compounds

RK424 was purchased from Enamine Ltd (Kiev, Ukraine). RK424 analogs were synthesized by Wakunaga Pharmaceutical Co. Ltd. (Akitakata, Japan) and a stock solution (10 mM) was prepared in dimethyl sulfoxide (DMSO). Oseltamivir phosphate was purchased from Sundia MediTech Company, Ltd (Shanghai, China) and a stock solution was prepared in DMSO. Leptomycin B (LMB) was purchased from Sigma (St. Louis, MO). LMB was dissolved in ethanol (EtOH) and then diluted in DMSO.

### Cells, transfection, and viruses

Madin-Darby canine kidney (MDCK) cells, HeLa cells, and human embryonic kidney 293T (HEK293T) cells from the repository of our laboratory, were grown in Dulbecco’s modified Eagle’s medium (DMEM, Invitrogen, Carlsbad, CA) containing Pen Strep Glutamine (PSG, GIBCO Industries Inc., Los Angeles, CA) and 10% fetal bovine serum (Sigma). Cells were cultured at 37°C in 5% CO_2_. Transfections were performed using FuGENE HD (Roche Diagnostics, Basel, Switzerland) according to the manufacturer's instructions. The influenza A viruses were propagated in MDCK cells at 37°C for 48 h in 5% CO_2_.

### Plasmid construction

The mammalian expression vector, pCAGGS, encoding monomeric red fluorescent protein (mRFP) fused to a Flag tag (mRFP-Flag), wild-type NP fused to mRFP-Flag tag (NP-mRFP-Flag), and wild-type NP fused to a Flag tag (NP-Flag) have been described previously [19,22]. The viral genome-expressing plasmids PB2/pHH21, PB1/pHH21, PA/pHH21, HA/pHH21, NP/pHH21, NA/pHH21, M/pHH21, NS/pHH21, and empty-pHH21, the expression plasmids PB1/pCAGGS, PB2/pCAGGS, PA/pCAGGS and NP/pCAGGS, and the vNP-luc/pHH21 plasmid (derived from the A/WSN/1933 (H1N1) virus) were kind gifts from Dr. Y Kawaoka (University of Tokyo). The plasmid encoding wild-type NP fused to a HA tag (NP-HA) was generated by polymerase chain reaction (PCR) using primers XhoI-NP (F), 5’-AAACTCGAGATGGCGACCAAAGGCACCAA-3’ and NotI-HA-NP(R), 5’-TAGCGGCCGCTTAAGCGTAATCTGGAACATCGTATGGGTAATTGTCGTACTCCTCTGCAT-3’ (restriction enzyme sites underlined) and NP/pCAGGS as a template. The PCR product was cloned into the XhoI and NotI sites within pCAGGS. The plasmid encoding wild-type NP fused to glutathione S-transferase (GST) (NP-GST) was generated by PCR using primers EcoRI-NP (F), 5’-GGGAATTCCATGGCGACCAAA-3’ and Xho1-GST-NP (R), 5’-AACTCGAGTTAATTGTCGTACTCCTCTGC-3’ (restriction enzyme sites underlined) and NP/pCAGGS as the template. The PCR product was cloned into the EcoRI and XhoI sites within pGEX6p-3 (GE Healthcare, Buckinghamshire, UK). Plasmids NP/pHH21 and NP/pCAGGS harboring site specific mutations (Y52H, R162A, S165A, L264A, Y289H, and Y487A) were created by Prime STAR Max DNA Polymerase (Takara Bio, Otsu, Japan) using the following primers Y52H-(F), 5’-AGTGATCATGAGGGACGGCTGATTCAG-3’ and Y52H-(R), 5’-TCCCTCATGATCACTGAGTTTAAGTTC-3’; R162A-(F), 5’-GATCCCGCGATGTGCTCACTGATGCAG-3’ and R162A-(R), 5’-GCACATCGCGGGATCCATTCCTGTGCG-3’; S165A-(F), 5’-ATGTGCGCACTGATGCAGGGCTCAACC-3’ and S165A-(R), 5’-CATCAGTGCGCACATCCTGGGATCCAT-3’; L264A-(F), 5’-TCTGCAGCCATATTGAGAGGGTCAGTT-3’ and L264A-(R), 5’-CAATATGGCTGCAGACCGTGCTAAAAA-3’; Y289H-(F), 5’-AGTGGACACGACTTTGAAAGAGAGGGA-3’ and Y289H-(R), 5’-AAAGTCGTGTCCACTGGCTACGGCAGA-3’; and Y487A-(F), 5’-GGATCTGCTTTCTTCGGAGACAATGCA-3’ and Y487A-(R), 5’-GAAGAAAGCAGATCCTTCATTACTCAT-3’ (point mutated sites underlined).

A pCAGGS plasmid encoding a Flag-tagged mutant NP (mutant NP-Flag) harboring all R162A, S165A, L264A, and Y487A mutations was generated using the method described above. All mutations were confirmed by DNA sequencing.

### *In vitro* antiviral activity assay

The antiviral activity of the different compounds was measured in a plaque assay as described previously [26]. Briefly, MDCK cells were inoculated with IAV at a multiplicity of infection (MOI) of 0.008. After washing, the cells were incubated in 2 mL of minimum essential medium (MEM) (2 ×) containing 1% agarose, 1 μg/mL trypsin (Worthington Biochemical Corporation), and each of the test compounds. Cells were then incubated at 37°C for 48 h in a 5% CO_2_ incubator. The number of plaques was counted and the IC_50_ calculated.

### Cell toxicity assay

Cell toxicity was measured in a water soluble tetrazolium salt-1 (WST-1) (Takara Bio) assay as previously described [26]. The 50% cytotoxic concentration (CC_50_) was calculated by comparing the viability of RK424-treated cells with that of DMSO-treated cells.

### Ethics statement

All animal experiments were approved by RIKEN Institutional Animal Use and Care Administrative Advisory Committees and were performed in accordance with RIKEN institutional guidelines and regulations (Certificate number: H23-2-101). All procedures were performed under pentobarbital treatment (30–50 mg/kg), and all efforts were made to minimize suffering. Mice were euthanized at the endpoint (14 days after virus inoculation) by intraperitoneal injection of pentobarbital (more than three times the dosage of pentobarbital for anesthesia). Mice were monitored and their body weight were measured daily during the study. Mice were euthanized if rapid and lasting weight loss (loss of more than 20% of body weight in a few days) was observed (a humane endpoint).

### *In vivo* antiviral activity assay

RK424 was dissolved in polyethylene glycol 400 (PEG400; Sigma) and then diluted with PBS to a final concentration of 1 mg/mL or 2 mg/mL. Six-week-old Balb/c mice (CREA Japan Inc, Tokyo, Japan) were infected intranasally with ten 50% lethal doses (LD_50_) of A/WSN/1933 (H1N1). Mice (eight per each RK424 treated-group) were intraperitoneally injected with RK424 2 h before virus exposure and then twice per day (2 × 5 mg/kg and 2 × 10 mg/kg) for 5 days beginning on the day of infection. Seven mice per positive control group also received 2 × 10 mg/kg of oseltamivir phosphate per day. Seven mice per negative control group received PBS. Bodyweight was measured daily and survival evaluated. Three mice from the negative control group and the 10 mg/kg RK424 group were euthanized on day 6 post-infection and virus titers in the lungs were measured in a plaque assay. Serial diluted oseltamivir phosphate was administered to three mice per each group (2 × 0.01, 2 × 0.03, 2 × 0.1, 2 × 0.3 and 2 × 1 mg/kg oseltamivir phosphate) twice per day according to the same protocol mentioned above and appropriate doses of oseltamivir phosphate were estimated for co-administration with RK424. In the co-administration study, 2 × 0.02 mg/kg oseltamivir phosphate together with 2 × 5 mg/kg or 2 × 10 mg/kg of RK424 were also administered twice per day in the same way and synergetic antiviral effect was evaluated with respect to mono therapy (0.02 mg/kg of oseltamivir phosphate or 5 or 10 mg/kg RK424) and the negative control group (PBS). Seven mice per each group were tested in the co-administration study.

### Plasma concentration and pharmacokinetic parameters of RK424

These studies were performed at Nemoto Science Co., Ltd. (Tsukuba, Japan). RK424 sufficient to achieve 1 mg/kg was dissolved in *N*,*N*- Dimethylacetamide (Wako Pure Chemical Industries, Ltd., Osaka, Japan) and isotonic sodium chloride solution (Otsuka Pharmaceutical Factory, Inc., Naruto, Japan) and was intravenously administrated; RK424 sufficient to achieve 10 mg/kg was dissolved in PEG 400 (Sigma) and was intraperitoneally administrated; and RK424 sufficient to achieve 10 mg/kg was dissolved in 0.5% methyl cellulose (Wako Pure Chemical Industries, Ltd.) solution and was orally administrated to eight-week-old Balb/c mice (CHARLES RIVER LABORATORIES JAPAN, INC., Yokohama, Japan). Three mice were assigned to each experimental group. Venous blood samples were collected at 0, 0.25, 0.5, 1, 2, 4, 6 and 24 h after intraperitoneal administration and collected at 0, 0.25, 0.5, 1, 2, 6 and 24 h after intravenous and oral administration. The blood samples were deproteinized and subjected to LC/MS/MS (ACQUITY UPLC system and Xevo TQ MS; Nihon Waters K.K., Tokyo, Japan) analyses. Pharmacokinetic parameters (Cmax, Tmax, AUC_0–24 h_, t_1/2_, Vd, and relative bioavailability) were calculated using Winnolin Ver.6.1 (Pharsight Corporation, CA).

### Plasma protein binding of RK424

These studies were performed at Wakunaga pharmaceutical Co., Ltd. (Akitakata, Japan).

Mouse plasma was prepared from seven-week-old mouse blood samples (CRJ, Yokohama, Japan). RK424 (50 μM) solution was prepared by mixing 5 mM RK424 in DMSO with mouse plasma and incubating the mixture at 37°C for 30 min. RK424 free from plasma protein was separated by centrifugal filtration using 10 kDa size cut filter (MultiScreen Ultracel-10; Merck Millipore, Darmstadt, Germany). The filtrate sample was measured by ACQUITY Ultra Performance Liquid chromatography (UPLC) system (Waters, Milford, MA) and the plasma protein binding rate was calculated.

### Mini-genome assay

HEK293T cells were cultured in 24 well plates at 37°C for 24 h. Cells were transfected with plasmid (PB2/pCAGGS, PB1/pCAGGS, PA/pCAGGS, or NP/pCAGGS) DNA (0.5 μg) and a luciferase RNA expression (vNP-luc/pHH21) vector. Cells were treated (or not) with the test compounds and luciferase activity was measured according to manufacturer’s instructions after incubation at 37°C for 48 h.

### Generation of recombinant viruses

Recombinant viruses were generated by DNA transfection as described previously [19]. Briefly, a co-culture of MDCK (4 × 10^5^) and HEK-293T (6 × 10^5^) cells was transfected with the eight viral genome-expressing plasmids or with the four viral protein-expressing plasmids. At 72 h post-transfection, recombinant viruses were harvested and the virus titer in the cell supernatants was determined in a plaque assay.

### Indirect immunofluorescence

HeLa cells were seeded on coverslips and grown to 60–70% confluence. Cells were then transfected with 0.5 μg of NP/pCAGGS or infected with A/WSN/1933 (H1N1) virus at an MOI of 10 in the presence or absence of the test compounds. After 48 h of transfection or 6 h of infection, cells were fixed with 4% paraformaldehyde for 20 min at room temperature (RT) and then further fixed with cold methanol for 10 min at −20°C. Cells were washed three times with PBS and then incubated with a primary anti-NP monoclonal antibody (MAb) (Santa Cruz Biotechnology Inc., Santa Cruz, CA; diluted 1:50 in PBS) for 1 h at RT. The cells were then washed with PBS and incubated with an Alexa Fluor 488-conjugated goat secondary antibody against rabbit IgG (H+L, Invitrogen: 1:500 in PBS) for 1 h at RT. The cells were then incubated with PBS containing Hoechst 33342 for 5 min and then washed three times with PBS. The excitation and emission wavelengths were set at 488 nm and 519 nm, respectively. Prepared samples were observed under a confocal laser scanning microscope (FV 1000; Olympus, Tokyo, Japan).

### Nuclear export assay

HeLa cells were attached to glass cover slips at 37°C for 24 h and then transiently transfected with NP-mRFP-Flag/pCAGGS. After 48 h, cells were washed twice with cold transport buffer (20 mM HEPES (pH 7.4), 110 mM potassium acetate, 2 mM magnesium acetate, 1 mM EGTA (pH 7.4), 2 mM DTT, 1 μg/mL aprotinin, 1 μg/mL leupeptin, and 1 μg/mL pepstatin) and permeabilized with digitonin (50 μg/mL; Sigma) in transport buffer for 5 min on ice. The cells were then washed with cold transport buffer and incubated with apyrase (10 U/mL; Sigma) for 5 min on ice. After washing twice with cold transport buffer, the cells were incubated in cold transport buffer for 10 min on ice before addition of a the reaction mixture (40 μg HeLa cell lysate, 10 mg/mL bovine serum albumin (BSA), 10 mM ATP, 5 mM creatine phosphate, 0.64 U creatine kinase) containing (or not) the test compounds. The mixture was the incubated at 30°C for 1 h. After washing three times with cold transport buffer, mRFP fluorescence was observed under a confocal laser scanning microscope (FV 1000; Olympus). The fluorescence intensity in the nuclei was analyzed with MetaMorph software (Molecular Devices Inc., Downingtown, PA). The fluorescence intensity of NP-mRFP-Flag was quantified in >100 nuclei in each of three independent experiments.

### Purification of NP proteins fused to different tags

HEK293T cells were transfected with 10 μg of NP-Flag/pCAGGS, NP-HA/pCAGGS, NP-mRFP-Flag/pCAGGS, or mRFP-Flag/pCAGGS for 48 h. The cells were then washed with PBS and lysed in 500 μL of lysis buffer (10 mM Tris-HCl (pH 7.8), 150 mM NaCl, 1 mM EDTA and 1% NP-40) at 4°C for 1 h. The lysates were centrifuged at 20,000 × g for 10 min and the supernatants collected and mixed with ANTI-FLAG M2 agarose beads (Sigma) or ANTI-HA agarose beads (Sigma). After incubating at 4°C for 24 h, the beads were washed three times with wash buffer (10 mM Tris-HCl (pH 7.4), 150 mM NaCl, and 0.05% NP-40). The proteins were purified by adding 3 × Flag peptide (Sigma) or HA peptide (Sigma). The purified NP-Flag and NP-HA proteins were then subjected to 10% SDS-PAGE and analyzed by western blotting. FLAG agarose beads coupled to mRFP-Flag, NP-mRFP-Flag, and NP-Flag-bound were used to analyze the interactions between NP and CRM1, or between NP and NP, before elution with the 3 × Flag peptide.

### Photo-cross-linked small-molecule RK424 affinity beads

RK424 was cross-linked to Sepharose beads as previously described [32]. Briefly, *N*-hydroxysuccinimide-activated beads were washed three times with 1 mM aqueous HCl followed by coupling solution (0.1 M NaHCO_3_ and 50% dioxane mixture). A solution of photoaffinity linker in coupling solution was then added to the beads, which were then incubated at 37°C for 2 h on a rotor. After washing five times with coupling solution, the beads were blocked with 1 M ethanolamine in 0.1 M Tris–HCl (pH 8.0) buffer at 37°C for 1 h on a rotor. The beads were then placed in a spin column and washed three times with Milli-Q water and methanol before being transferred to a glass sample vial. A methanol solution containing RK424 was then added to the beads and the mixture concentrated and dried *in vacuo*. The beads were then irradiated in a UV cross-linker at 365 nm (4 J/cm^2^) and washed with methanol to yield RK424-cross-linked affinity beads. Purified NP-Flag proteins were added to 20 μL of RK424-cross-linked affinity beads or uncross-linked beads. After incubating at 4°C for 24 h, the beads were washed five times with wash buffer and bound proteins eluted in 10% SDS-PAGE sample buffer at 100°C for 5 min. The proteins that bound to RK424 were then separated in 10% SDS–PAGE gels and detected by western blotting with an anti-Flag M2 MAb (Sigma).

### Binding of CRM1 to FLAG agarose beads coupled to NP-Flag protein (NP-Flag beads)

NP-Flag beads were incubated with HeLa cell lysate and different concentrations of RK424. After incubation at 4°C for 24 h, the NP-Flag beads were washed five times with wash buffer and the bound proteins separated in 10% SDS-PAGE gels. Binding of CRM1 to NP-Flag beads was detected by western blotting with an anti-CRM1 MAb (BD Biosciences, Franklin Lakes, NJ). LMB was used as positive control (LMB inhibits the binding of NP to CRM1). EtOH and DMSO were used as vehicle controls for LMB and RK424, respectively.

### Surface plasmon resonance (SPR) analysis of the NP-RNA interaction

A biotinylated 2-O-methylated RNA oligonucleotide (5-UUU GUU ACA CAC ACA CAC GCU GUG-3) was purchased from Hokkaido System Science CO., Ltd. (Sapporo, Japan) and immobilized on an streptavidin-coated sensor chip (GE Healthcare) at a surface density of 100 resonance units (RU), according to manufacturer’s instructions The inhibitory effect of RK424 to NP-RNA binding was examined by incubating 10 ng/μL NP-FLAG proteins with RK424 at RT for 1 h. The RK424-treated samples were then injected onto the sensor chip surface and the SPR signal measured in a Biacore T-100 (GE Healthcare). Samples were injected in running buffer (200 mM NaCl, 20 mM Tris-HCl (pH 7.4), and 0.05% Tween 20) at 25°C.

### Analysis of the NP-NP interaction

FLAG agarose beads coupled to mRFP-Flag (mRFP-Flag beads), NP-mRFP-Flag (NP-mRFP-Flag beads), and purified NP-HA proteins were treated with RNase A (final concentration of 200 μg/ml) at RT for 10 min followed by a further incubation at 4°C overnight. The purified NP-HA proteins were then incubated with RK424 at 4°C for 1 h. Purified NP-HA proteins were then added to NP-mRFP-Flag beads or mRFP-Flag beads and incubated overnight at 4°C. The incubated beads were washed five times with wash buffer and then subjected to 10% SDS-PAGE. Binding of NP-HA to mRFP-Flag or NP-mRFP-Flag was detected by western blotting with an anti-HA Mab (MBL, Nagoya, Japan). An anti-Flag M2 Mab (Sigma) was used as a loading control.

### *In vitro* transcription and vRNA synthesis

vRNA encoding the M gene was generated using a RiboMAX Large Scale RNA Production System-T7 (Promega, Medison, WI). Briefly, approximately 1027 bp of the A/WSN/1933 (H1N1) virus negative-sense segment 7 genome was amplified from an M/pHH21 plasmid by PCR and the products purified using a QIAquick PCR purification kit (Qiagen, Valencia, CA). Five μg of the purified PCR product were added to a reaction mixture containing transcription buffer, rNTP mix (25 mM ATP, CTP, GTP and UTP), and T7 enzyme mix (final volume 100 μL) and incubated at 37°C for 4 h. Synthesized vRNA was treated with 5 units of RQ1 RNAse-Free DNase I (Promega) at 37°C for 15 min. The following primers were used to amplify the negative-sense segment 7 genome: T7_WSN_seg7-(F), 5’-GACTCAGTTAATACGACTCACTATATAGTTTTTTACTCCAGCTCTATGTTG-3’ and T7_WSNseg7-(R), 5’-AGCAAAAGCAGGTAGATATTG-3’ (T7 promoter sequence underlined).

### Size exclusion chromatography to assess oligomerization

*E*. *coli* strain BL21 CodonPlus (DE3)-RIL (Stratagene, La Jolla, CA) harboring the GST-NP/pGEX6p-3 plasmid was cultured in the presence of 1 mM isopropyl-b-D-1 thiogalactopyranoside (IPTG) for 10 h at 22°C and then collected by centrifugation. The cell pellets were resuspended and sonicated in lysis buffer (50 mM Tris-HCl (pH 7.4), 200 mM NaCl, 1% Triton X-100, 1 mg/mL lysozyme, and 0.15 mg/mL RNAse A). Glutathione Sepharose 4 Fast Flow beads (GSH beads; GE Healthcare) were then added to the cell lysate and rotated overnight at 4°C. After washing the beads, bound proteins were released from the beads using PreScission Protease (GE Healthcare). The GST-cleaved NP proteins were then incubated with Heparin Sepharose 6 Fast Flow beads (GE Healthcare) to dissociate RNA from NP. The bound NP proteins were then eluted with elution buffer (1.5 M NaCl and 50 mM Tris-HCl (pH 7.4)). The eluted samples were concentrated to 1 mg/mL and dialyzed into lower salt buffer (100 mM NaCl and 50 mM Tris-HCl (pH 7.4)) using an Amicon Ultra 50K device (Merck Millipore). The buffer exchanged proteins were maintained at 4°C to allow the equilibrium to shift from oligomers to monomers. The prepared NP proteins were then rotated with RK424 and vRNA at RT for 60 min. Samples were then loaded onto a Superdex 200 Increase 10/300 GL Column (GE Healthcare) attached to an ÄKTA purifier chromatography system (GE Healthcare). The size distribution data and integrated peak areas were calculated using the instruments software (UNICORN ver. 5.1; GE Healthcare).

### RNA FISH analysis

RNA FISH was performed according as previously described [26,29]. The probes targeting the PB2 segment were designed by STELLARIS RNA FISH PROBE DESIGNER and purchased from Biosearch Technologies, Inc. (Novato, CA). One set of probes contained a blend of 48 oligos, which comprised 20 mer single-stranded DNAs, each labeled with Quasar 670. The primer sequences are listed in S3 Table. MDCK cells (2 × 10^5^ cells/well) were seeded onto coverslips in 12-well plates and incubated overnight at 37°C. At 6 h post-infection with A/WSN/1933 (H1N1) at an MOI of 5 in the absence or presence of RK424, the cells were washed once with PBS and then fixed with 4% paraformaldehyde in PBS for 10 min at RT. After a brief wash with PBS, the cells were permeabilized in 0.5% Triton X-100 in PBS for 1 min at RT. Prior to hybridization, the cells were washed with PBS and incubated for 5 min in 2 × SSC (300 mM sodium chloride, 30 mM sodium citrate) containing 10% formamide. To detect viral RNAs, 2 μM of labeled probes in 15 μl of hybridization buffer (10% dextran sulfate, 2 mM vanadyl ribonucleoside complexes (VRC; New England BioLabs), 0.02% BSA, 50 mg *E*. *coli* tRNA, 2 × SSC, and 10% formamide) was added to each sample. Hybridization was performed in a humidified chamber maintained at 37°C for 16 h. The samples were then washed twice for 30 min at 37°C with 10% formamide containing 2 × SSC supplemented with 2 mM VRC. Nuclei were stained with Hoechst 33342 and the cells were washed with PBS. Prepared samples were observed under a confocal laser scanning microscope (FV 1000; Olympus).

### Generation of escape viruses by *in vitro* passaging an IAV in the presence of antiviral compound

MDCK cells were cultured in 12 well plate at 37°C for 24 h. MDCK cells were infected with A/WSN/1933 (H1N1) strain at 100 PFU/well and cultured in the presence of serial dilutions of RK424 or nucleozin. Viral replication was monitored for one week to observe any cytopathic effect (CPE) in the culture plates. The supernatants were collected from wells treated with the highest concentration of compound in wells in which CPE was observed and then stored at -80°C for subsequent analysis. Virus was serially passaged by using 5 μL of viral stock from the preceding passage to infect fresh MDCK cells in the presence of increasing concentrations of compound. The compound concentrations used in the selection varied, depending on the highest concentration of compound at which CPE was observed in the preceding passage. These passages led to the generation of mutant viruses resistant to RK424 or nucleozin when mutations occurred in the viral protein targeted by the antiviral compound. The selection was carried out for a total of four passages with RK424 or nucleozin. RK424 concentrations ranged from 0.1–2 μM at 1^st^ passage, 0.2–3 μM at 2^nd^ passage, 0.3–3μM at 3^rd^ passage and 0.4–3 μM at 4^th^ passage. Nucleozin concentrations ranged from 0.1–2 μM at 1^st^ passage, 0.3–3 μM at 2^nd^ passage, 1–5 μM at 3^rd^ passage and 3–10 μM at 4^th^ passage. After the 4^th^ passage, drug sensitivity was confirmed by using the plaque reduction assay with 10 μM RK424 or nucleozin as mentioned above.

### Sequence analysis of the NP coding regions in the escape mutant

Viral RNA was isolated from the plaque-purified viruses at 3^rd^ passage with RK424 and 4^th^ passage with nucleozin using Trizol LS reagent (Invitrogen) according to the manufacturer’s protocol. Reverse transcription (RT)-PCR was carried out with the SuperScript III First-Strand Synthesis System for RT-PCR (Invitrogen) according to the manufacturer’s protocol using a minus-strand-specific universal primer; Uni12 (F), 5’-agcaaaagcagg-3’. The coding region of the NP sequence in the RT product was amplified with the above described EcoRI-NP (F) and Xho1-GST-NP (R) primers by using KOD-Plus-(Toyobo, Osaka, Japan) according to the manufacturer’s protocol. The PCR-amplified product was directly sequenced on an ABI3730xl DNA Analyzer using an ABI PRISM BigDye Terminator v 3.1 Ready Reaction Cycle Sequencing Kit (Applied Biosystems, Foster City, CA).

### Calculation of the conservation ratio

Complete sequences for IAV NP were downloaded using the advanced database search at the NCBI’s Influenza Virus Resource [53]. Avian, human, and swine sequences were obtained using the following parameters: type A, full length only, and whole collection date. The MAFFT (version 6) program was used to align the sequences from both groups using a FFT-NS-2 strategy [54]. The 3,759 human-, 3,273 avian-, and 651 swine-origin NP sequences were then used to calculate the amino acid conservation ratio.

### Statistical analysis

The two-tailed t-test and the log-rank test were used for statistical analysis. P-values <0.05 were considered statistically significant.

### Docking analysis of RK424 and NP

*In silico* analysis of RK424 and NP binding was performed using AutoDock (version 4.2) [31]. The steric structure of monomeric influenza A/WSN/1933 (H1N1) NP was derived from a NP multimer structure deposited in the PDB (PDB ID: 2IQH). The NP structure was pre-processed, i.e., hydrogen atoms were added prior to docking simulation. During docking simulation, the optimal conformations for each ligand-receptor binding state were retreated in spacious searching space. Subsequent analysis of the interaction between RK424 and NP was conducted using MOE version 2013.0801 [55].

### Search for molecules with binding pockets structurally similar to the NP pocket

The pocket structures of NP and PA were extracted from complete NP (PDB ID: 2IQH) and PA (PDB ID: 4E5E) structures by removing residues within 10 Å of the small pocket (R162, S165, L264, and Y487) and the endonuclease motif (P107, D108, E119, and K134), respectively. Protein structure libraries (PoSSuM [38] or ProBiS [39]) were then searched for structures similar to the NP and PA pockets.

### Accession numbers

The accession numbers for genes and proteins mentioned in this study are shown below. PB2 (GenBank Accession No. J0217), PB1 (GenBank Accession No.J02178), PA (GenBank Accession No. X17336), HA (GenBank Accession No. J02176), NP (GenBank Accession No. CY010791), NA (GenBank Accession No. L25817), M (GenBank Accession No. L25818), NS (GenBank Accession No. M12597), firefly luciferase (GenBank Accession No. AAA89082.1), mRFP (GenBank Accession No. AF506027), and GST (GenBank Accession No. AAB37352).

## Supporting Information

S1 Fig Plasma concentration of RK424. RK424 (10 mg/kg) was intraperitoneally or orally administrated to 8-week-old Balb/c mice. The plasma concentration of RK424 was determined at 0, 0.5, 1, 2, 4, 6, and 24 h after intraperitoneal administration and at 0, 0.5, 1, 2, 6, and 24 h after oral administration by LC/MS/MS.
(TIF)

S2 Fig Estimation of appropriate dose of Oseltamivir phosphate (Os) for co-administration with RK424. Serial diluted Os was intraperitoneally administered to 6-week-old Balb/c mice 2 h prior to virus exposure and then twice per day for 5 days beginning on the day of infection. PBS was used as a negative control. Mice were infected intranasally with ten 50% lethal doses (LD50) of influenza A/WSN/1933 (H1N1) virus. (A) The body weight of three mice from each group was monitored and (B) survival rate was calculated.
(TIF)

S3 Fig Plaque formation after RK424 treatment. MDCK cells were infected with influenza A/WSN/1933 (H1N1) for 1 h at 37°C before being overlaid with agarose containing oseltamivir phosphate (Os) or RK424. Plaque formation was visualized by staining with crystal violet. Three independent experiments were performed and one representative result is shown.
(TIF)

S4 Fig Effect of RK424 on virus adsorption. Influenza A/WSN/1933 (H1N1) virus was exposed to RK424 for 1 h at 4°C. The virus was then used to infect MDCK cells in a plaque assay. The viral titer was calculated by counting of number of plaques formed by virus exposed to RK424 or dimethyl sulfoxide (DMSO) (control). Values represent the mean ± SD of three independent experiments.
(TIF)

S5 Fig Stage of the viral life cycle targeted by RK424. RK424 (0.5 μM or 2.0 μM) was added at the indicated time points. After 2 h treatment with RK424 at each time point, MDCK cells were washed with PBS and resuspended in fresh media. Cultured supernatants were collected 12 h post-infection. The viral titers of collected supernatants were estimated by the plaque titration assay. Values represent the mean ± SD of three independent experiments.
(TIF)

S6 Fig Three different models of RK424 binding to NP. *In silico* docking analysis identified three potential binding sites for RK424 on NP. The configuration of each binding site was visualized using PyMol. Functional domains located in close proximity to binding site 1 are colored orange (RNA binding groove: amino acid (aa) 1–180), yellow (NES3: aa 256–266), and purple (dimer interface: aa 482–489). The binding energy for each potential configuration of RK424 is shown in the table.
(TIF)

S7 Fig Models showing the binding of RK424 to NP binding site 1. The docking models depict six different RK424 configurations (0, 1, 2, 3, 5, and 6). The side chains of the amino acid residues involved in the NP-RK424 interaction are colored red. Functional domains close to binding site 1 are colored orange (RNA binding groove: amino acid (aa) 1–180), yellow (NES3: aa 256–266), and purple (dimer inter-face: aa 482–489). The binding interactions between the six RK424 configurations and the amino acid residues at binding site l are listed in the table.
(TIF)

S8 Fig Models showing the binding of RK424 to the potential NP binding site 2 and 3. The binding of RK424 configuration 4 to potential binding site 2, and of RK424 configuration 7 to potential binding site 3, is shown. The side chains of the amino acid residues involved in the NP-RK424 interaction are colored red. In contrast to the residues near to binding site 1 (S4 Fig), these amino acid residues are not located within known functional NP domains. The interactions between different RK424 configurations and the seven amino acid residues within binding sites 2 and 3, the mutant population, and the conservation ratio of these residues, are shown in the lower table. Perl script was used to analyze 7683 NP sequences derived from human, avian, and swine influenza A viruses, and the conservation ratio and mutant population were calculated.
(TIF)

S9 Fig Oligomerization assay incorporating purified NP proteins and vRNA synthesized by *in vitro* transcription. (A) NP proteins purified on GSH affinity beads were subjected to electrophoresis in 10% SDS-PAGE gels and the purity checked by Coomassie Brilliant Blue (CBB) staining. (B) vRNA (M segment) was synthesized by *in vitro* transcription and subjected to electrophoresis on an 0.8% agarose gel containing ethidium bromide. The vRNA was visualized under UV irradiation. (C) Size exclusion chromatogram showing different NP protein configurations under high salt buffer (300 mM NaCl; upper panel) and low salt buffer (100 mM NaCl; lower panel) conditions.
(TIF)

S10 Fig Structural similarity between the NP and PA pockets and pockets on other proteins. (A) The pocket structures of NP and PA were extracted from complete NP (PDB ID: 2IQH) and PA (PDB ID: 4E5E) structures by removing residues within 10 Å of the small pocket (R162, S165, L264, and Y487) and the endonuclease motif (P107, D108, E119, and K134), respectively. (B) The pocket structures of NP and PA (red) were compared with other potential pocket structures identified from the database (blue). (C) Significant hits (Z-score>1.96) against the PA pocket are shown together with the PDB ID, Chain ID, and Z-score.
(TIF)

S11 Fig Chemical structures of NP inhibitors. The structure of compound 3 (A) and nucleozin (B) are indicated. Compound 3 inhibits formation of the NP trimer by disrupting the salt bridge between E339 and R416 within the NP tail loop binding pocket [23] and nucleozin inhibits the formation of higher-order NP oligomers by cross-linking two NP molecules [24,25].
(TIF)

S1 Table PK (Pharmacokinetic) parameters of intravenously administered RK424. PK parameters (C_max_, C_free max_, T_max_, AUC_0–24 h_, t_1/2_, and Vd) were calculated using Winnolin Ver.6.1 and different plasma concentrations of RK424. Plasma protein binding was also measured by UPLC.
(TIF)

S2 Table PK parameters of intraperitoneally and oral administered RK424. PK parameters (C_max_, C_free max_, T_max_, AUC_0–24 h_, t_1/2_, and Vd) were calculated using Winnolin Ver.6.1 and different plasma concentrations of RK424. Relative bioavailability was also calculated using intraperitoneal and oral AUC_0–24 h_.
(TIF)

S3 Table Probe sequences used to target the vRNA coding viral PB2 protein in infected cells. The probes were designed by STELLARIS RNA FISH PROBE DESIGNER and purchased from Biosearch Technologies, Inc.
(TIF)
