# Supplementary figures and images for: A Novel Antiviral Target Structure Involved in the RNA Binding, Dimerization, and Nuclear Export Functions of the Influenza A Virus Nucleoprotein
Source: PLoS Pathog. 2015 Jul 29;11(7):e1005062. doi: 10.1371/journal.ppat.1005062 (PMC4519322; doi:10.1371/journal.ppat.1005062)

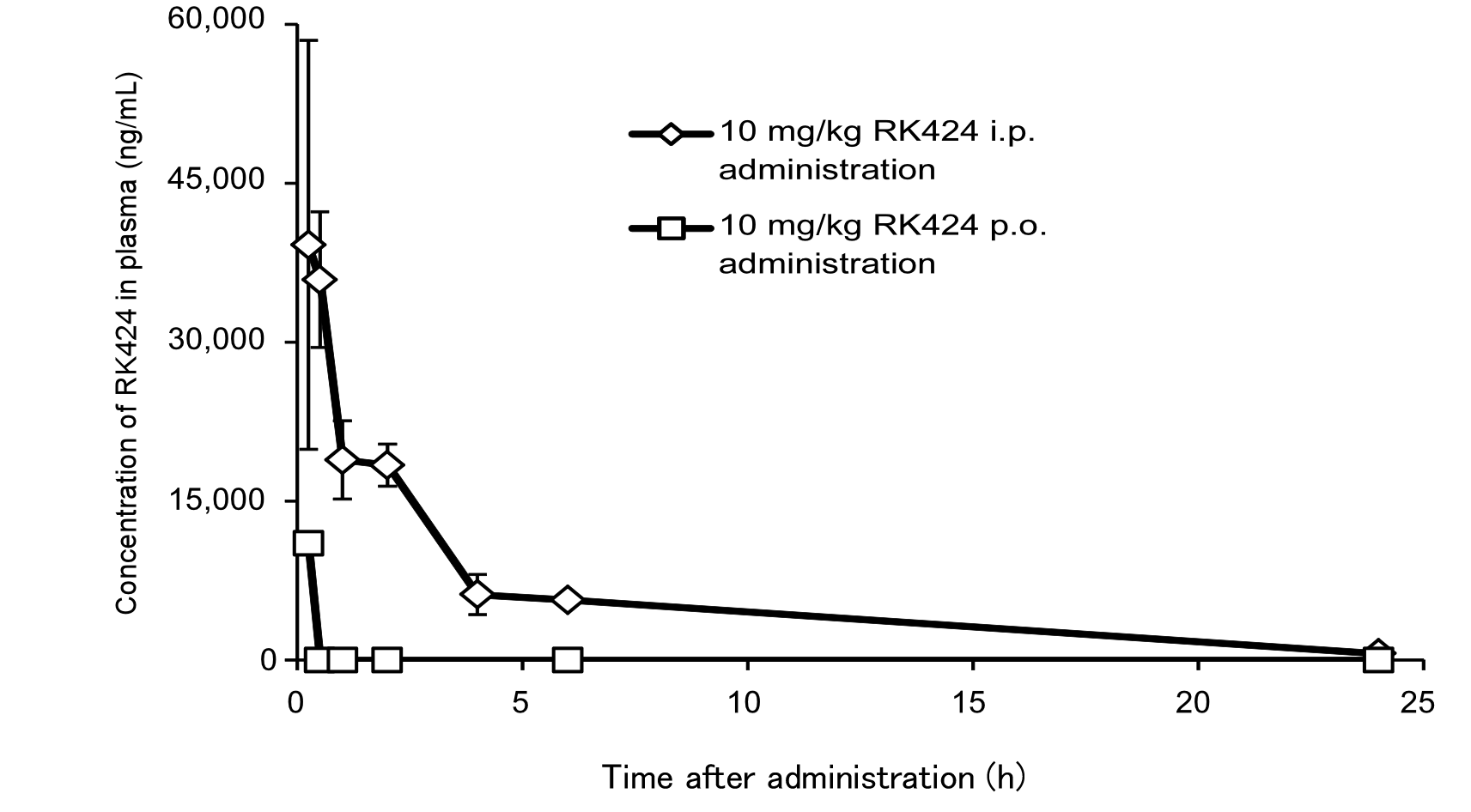

Supplement: S1 Fig — RK424 (10 mg/kg) was intraperitoneally or orally administrated to 8-week-old Balb/c mice. The plasma concentration of RK424 was determined at 0, 0.5, 1, 2, 4, 6, and 24 h after intraperitoneal administration and at 0, 0.5, 1, 2, 6, and 24 h after oral administration by LC/MS/MS. (TIF) [file ppat.1005062.s001.tif]

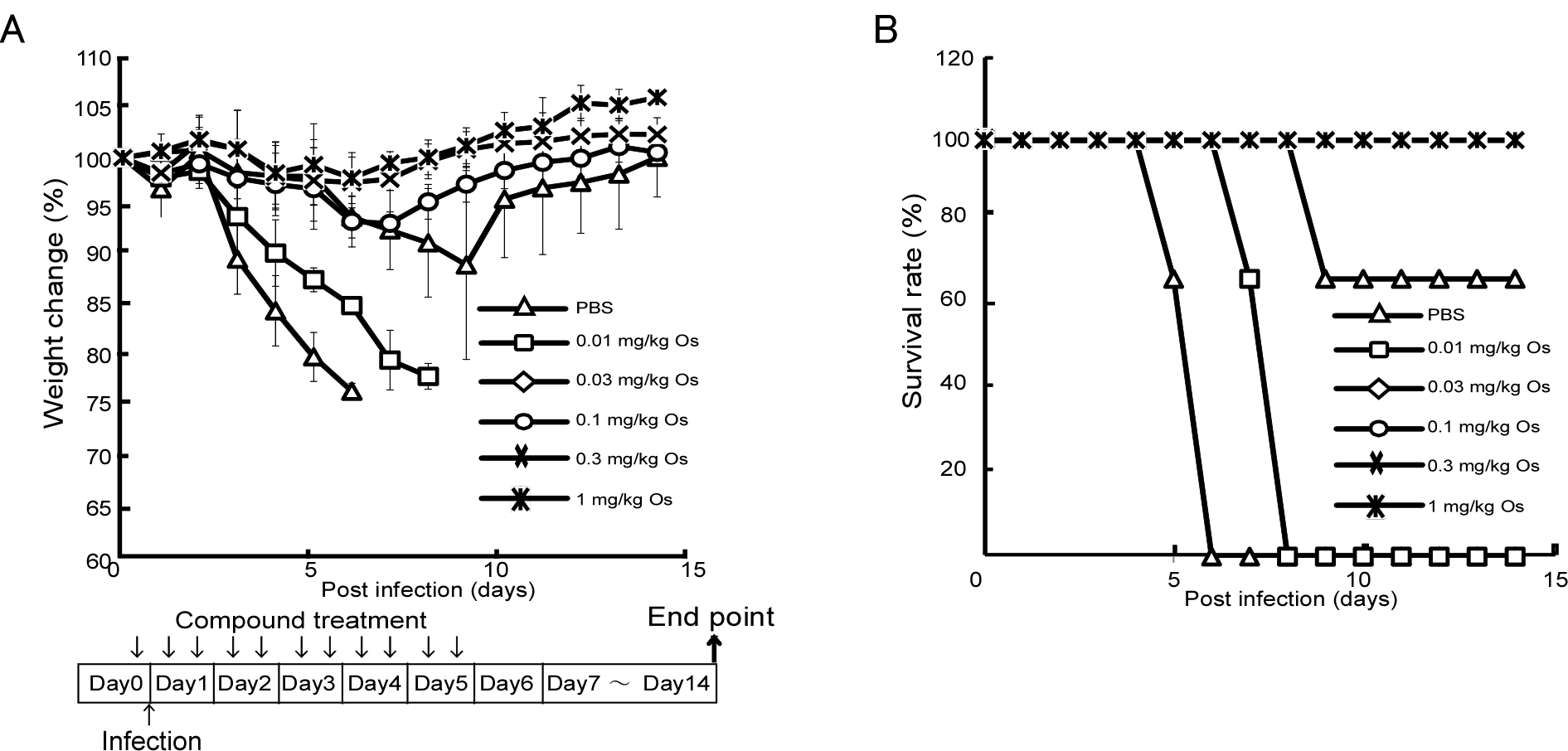

Supplement: S2 Fig — Serial diluted Os was intraperitoneally administered to 6-week-old Balb/c mice 2 h prior to virus exposure and then twice per day for 5 days beginning on the day of infection. PBS was used as a negative control. Mice were infected intranasally with ten 50% lethal doses (LD50) of influenza A/WSN/1933 (H1N1) virus. (A) The body weight of three mice from each group was monitored and (B) survival rate was calculated. (TIF) [file ppat.1005062.s002.tif]

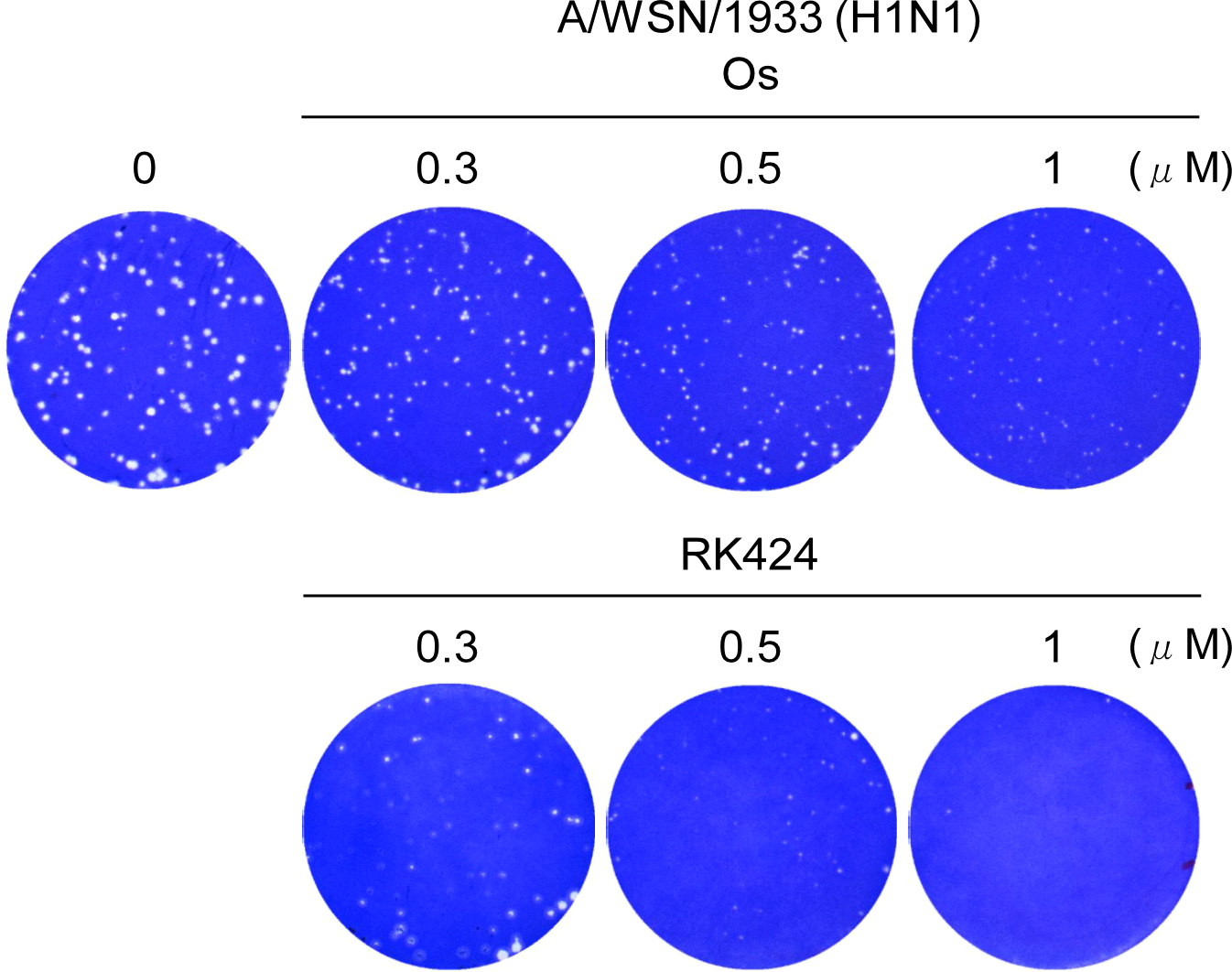

Supplement: S3 Fig — MDCK cells were infected with influenza A/WSN/1933 (H1N1) for 1 h at 37°C before being overlaid with agarose containing oseltamivir phosphate (Os) or RK424. Plaque formation was visualized by staining with crystal violet. Three independent experiments were performed and one representative result is shown. (TIF) [file ppat.1005062.s003.tif]

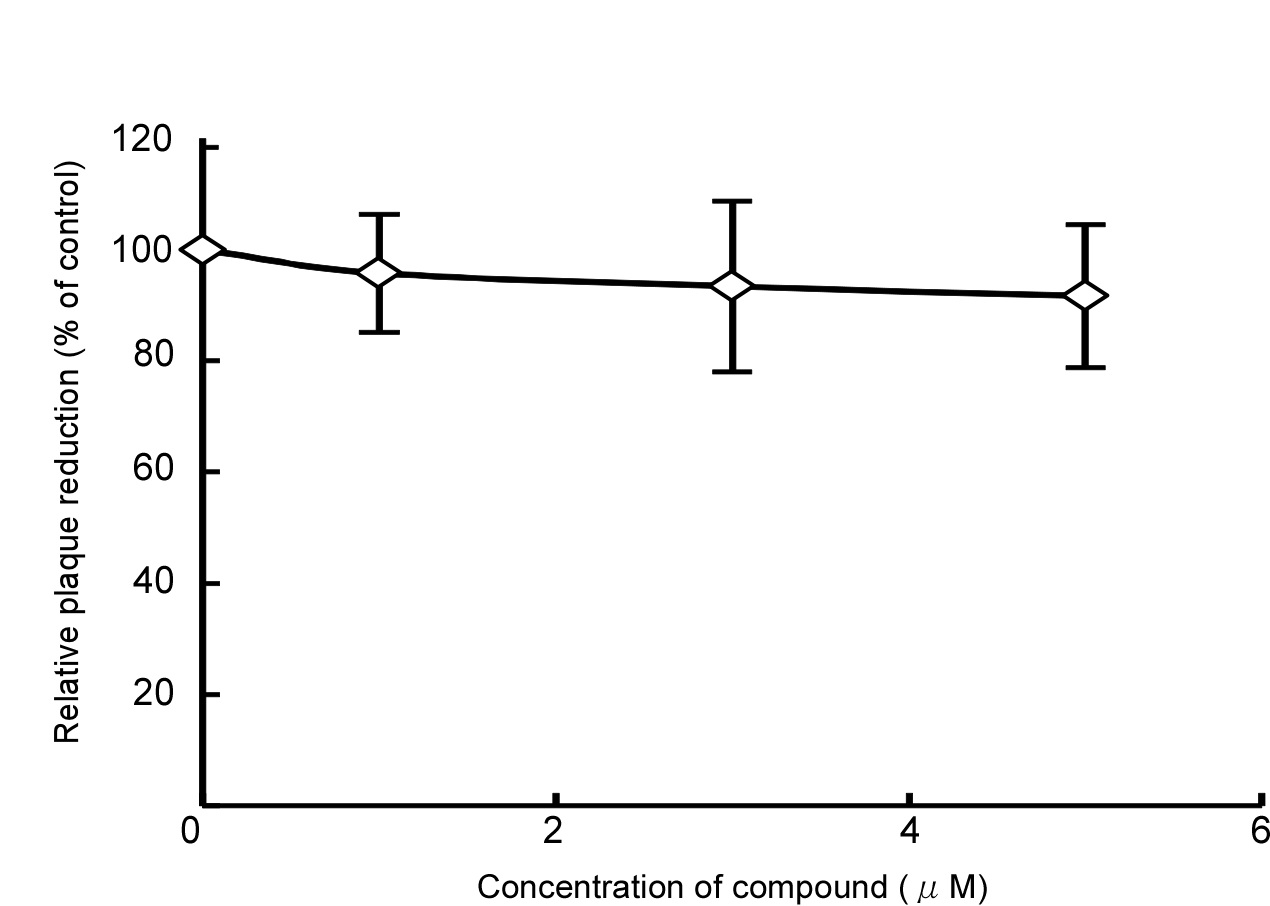

Supplement: S4 Fig — Influenza A/WSN/1933 (H1N1) virus was exposed to RK424 for 1 h at 4°C. The virus was then used to infect MDCK cells in a plaque assay. The viral titer was calculated by counting of number of plaques formed by virus exposed to RK424 or dimethyl sulfoxide (DMSO) (control). Values represent the mean ± SD of three independent experiments. (TIF) [file ppat.1005062.s004.tif]

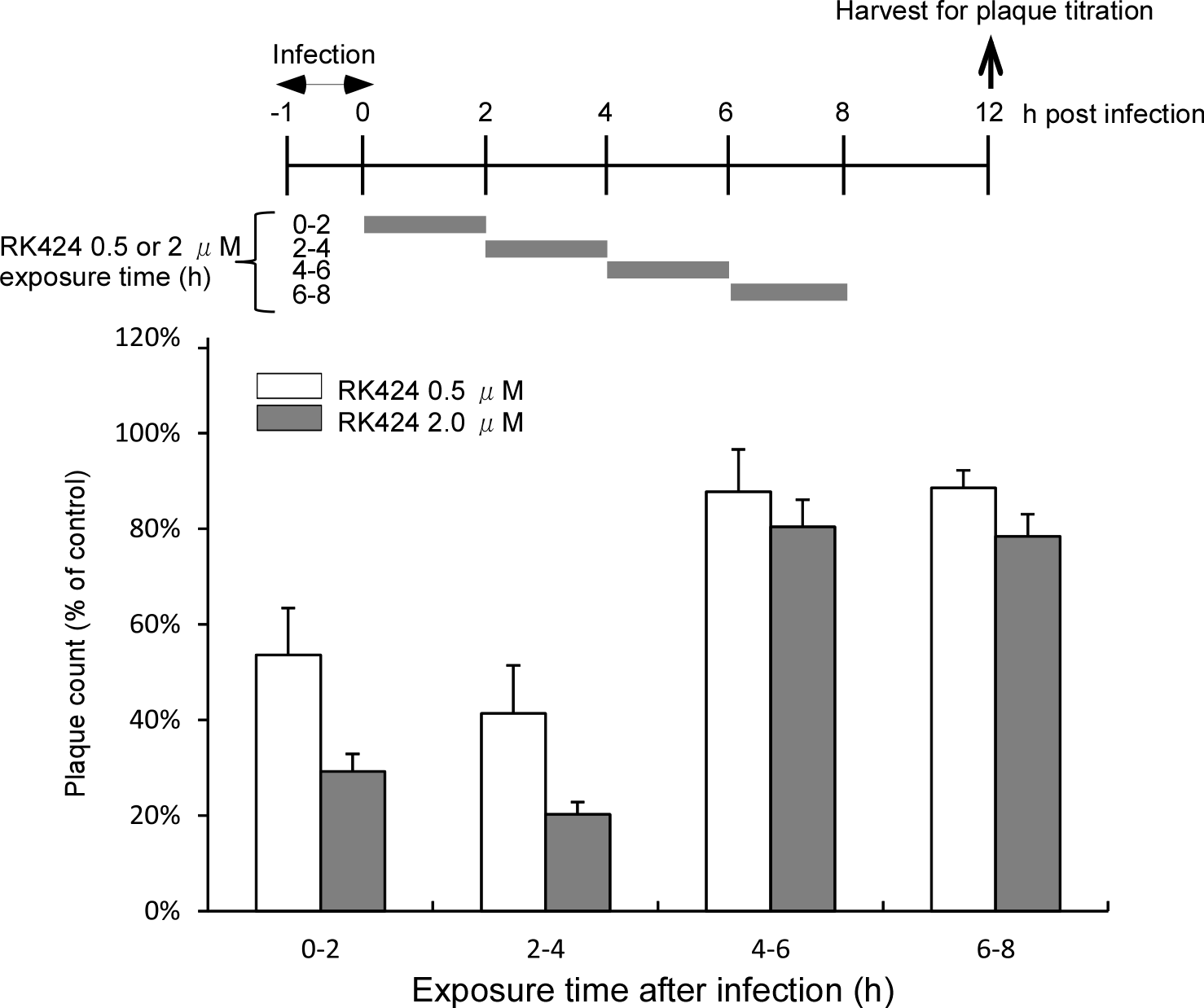

Supplement: S5 Fig — RK424 (0.5 μM or 2.0 μM) was added at the indicated time points. After 2 h treatment with RK424 at each time point, MDCK cells were washed with PBS and resuspended in fresh media. Cultured supernatants were collected 12 h post-infection. The viral titers of collected supernatants were estimated by the plaque titration assay. Values represent the mean ± SD of three independent experiments. (TIF) [file ppat.1005062.s005.tif]

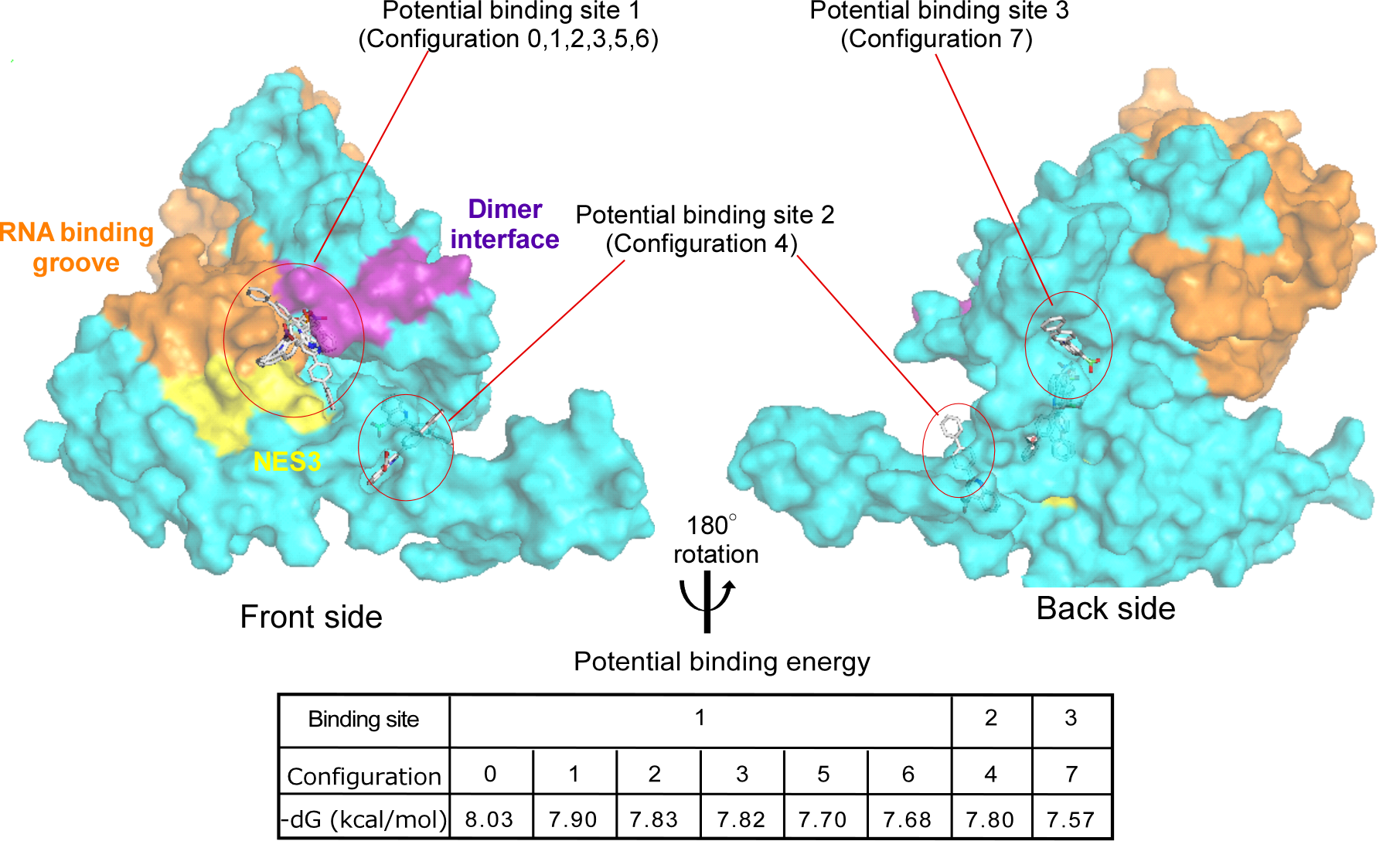

Supplement: S6 Fig — In silico docking analysis identified three potential binding sites for RK424 on NP. The configuration of each binding site was visualized using PyMol. Functional domains located in close proximity to binding site 1 are colored orange (RNA binding groove: amino acid (aa) 1–180), yellow (NES3: aa 256–266), and purple (dimer interface: aa 482–489). The binding energy for each potential configuration of RK424 is shown in the table. (TIF) [file ppat.1005062.s006.tif]

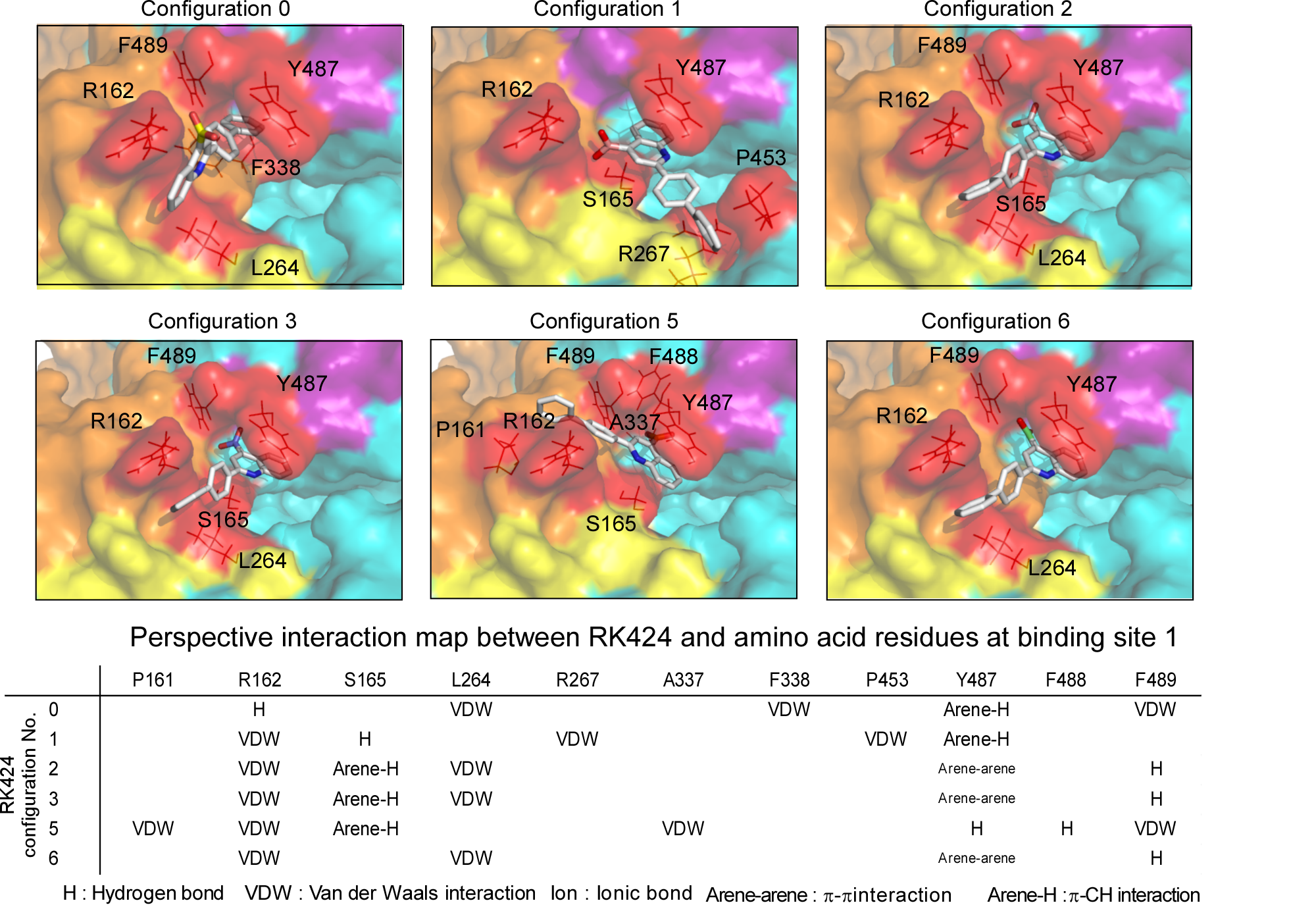

Supplement: S7 Fig — The docking models depict six different RK424 configurations (0, 1, 2, 3, 5, and 6). The side chains of the amino acid residues involved in the NP-RK424 interaction are colored red. Functional domains close to binding site 1 are colored orange (RNA binding groove: amino acid (aa) 1–180), yellow (NES3: aa 256–266), and purple (dimer inter-face: aa 482–489). The binding interactions between the six RK424 configurations and the amino acid residues at binding site l are listed in the table. (TIF) [file ppat.1005062.s007.tif]

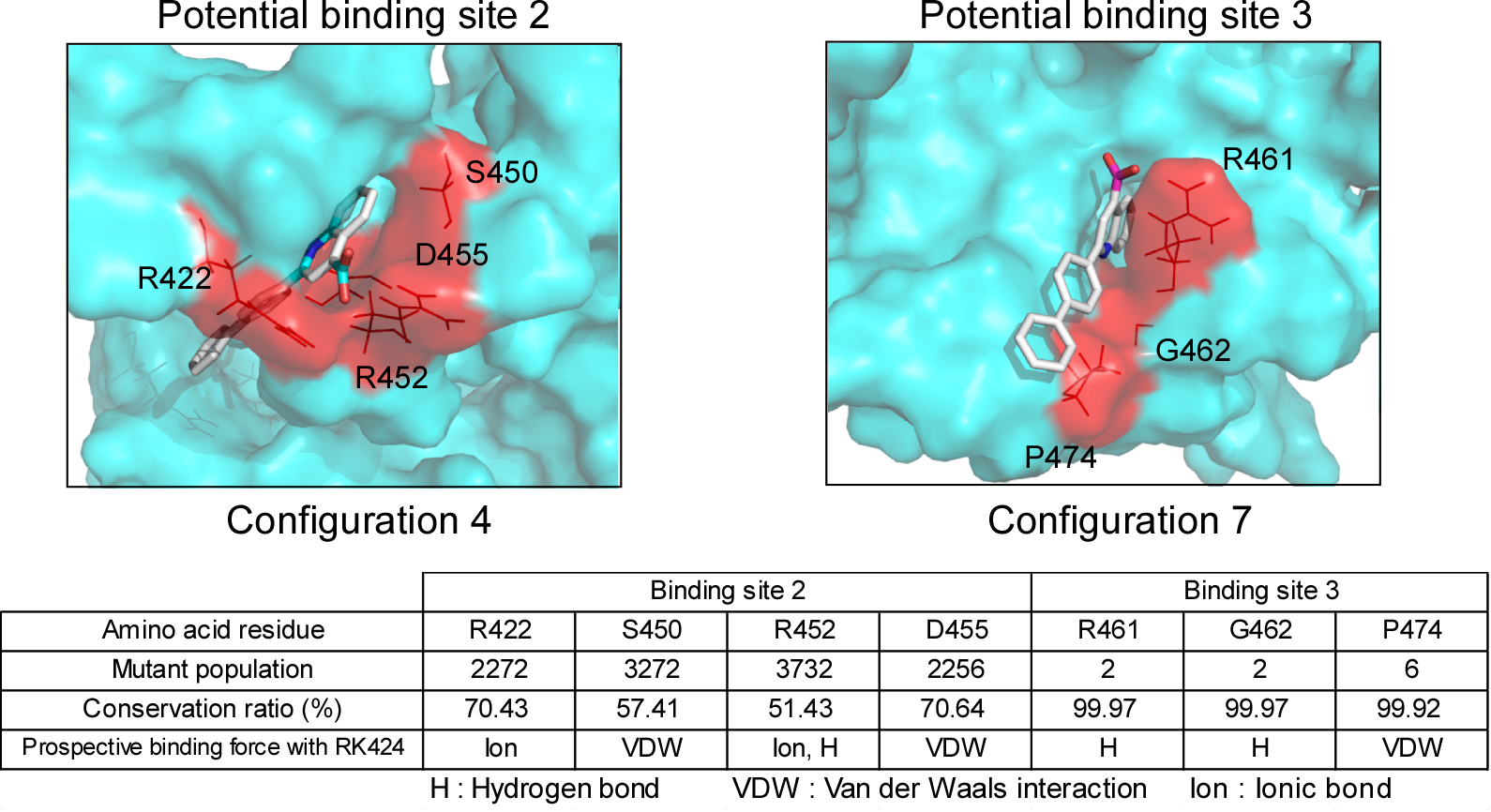

Supplement: S8 Fig — The binding of RK424 configuration 4 to potential binding site 2, and of RK424 configuration 7 to potential binding site 3, is shown. The side chains of the amino acid residues involved in the NP-RK424 interaction are colored red. In contrast to the residues near to binding site 1 (S4 Fig), these amino acid residues are not located within known functional NP domains. The interactions between different RK424 configurations and the seven amino acid residues within binding sites 2 and 3, the mutant population, and the conservation ratio of these residues, are shown in the lower table. Perl script was used to analyze 7683 NP sequences derived from human, avian, and swine influenza A viruses, and the conservation ratio and mutant population were calculated. (TIF) [file ppat.1005062.s008.tif]

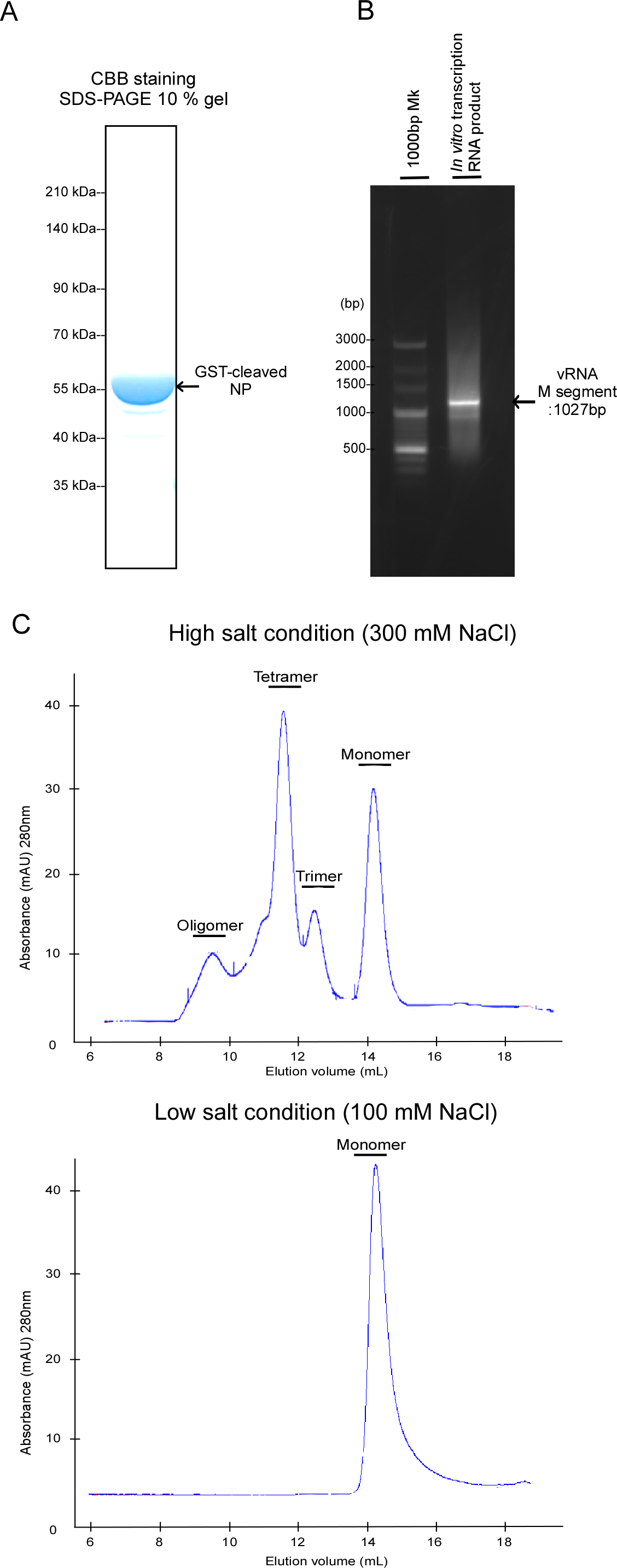

Supplement: S9 Fig — (A) NP proteins purified on GSH affinity beads were subjected to electrophoresis in 10% SDS-PAGE gels and the purity checked by Coomassie Brilliant Blue (CBB) staining. (B) vRNA (M segment) was synthesized by in vitro transcription and subjected to electrophoresis on an 0.8% agarose gel containing ethidium bromide. The vRNA was visualized under UV irradiation. (C) Size exclusion chromatogram showing different NP protein configurations under high salt buffer (300 mM NaCl; upper panel) and low salt buffer (100 mM NaCl; lower panel) conditions. (TIF) [file ppat.1005062.s009.tif]

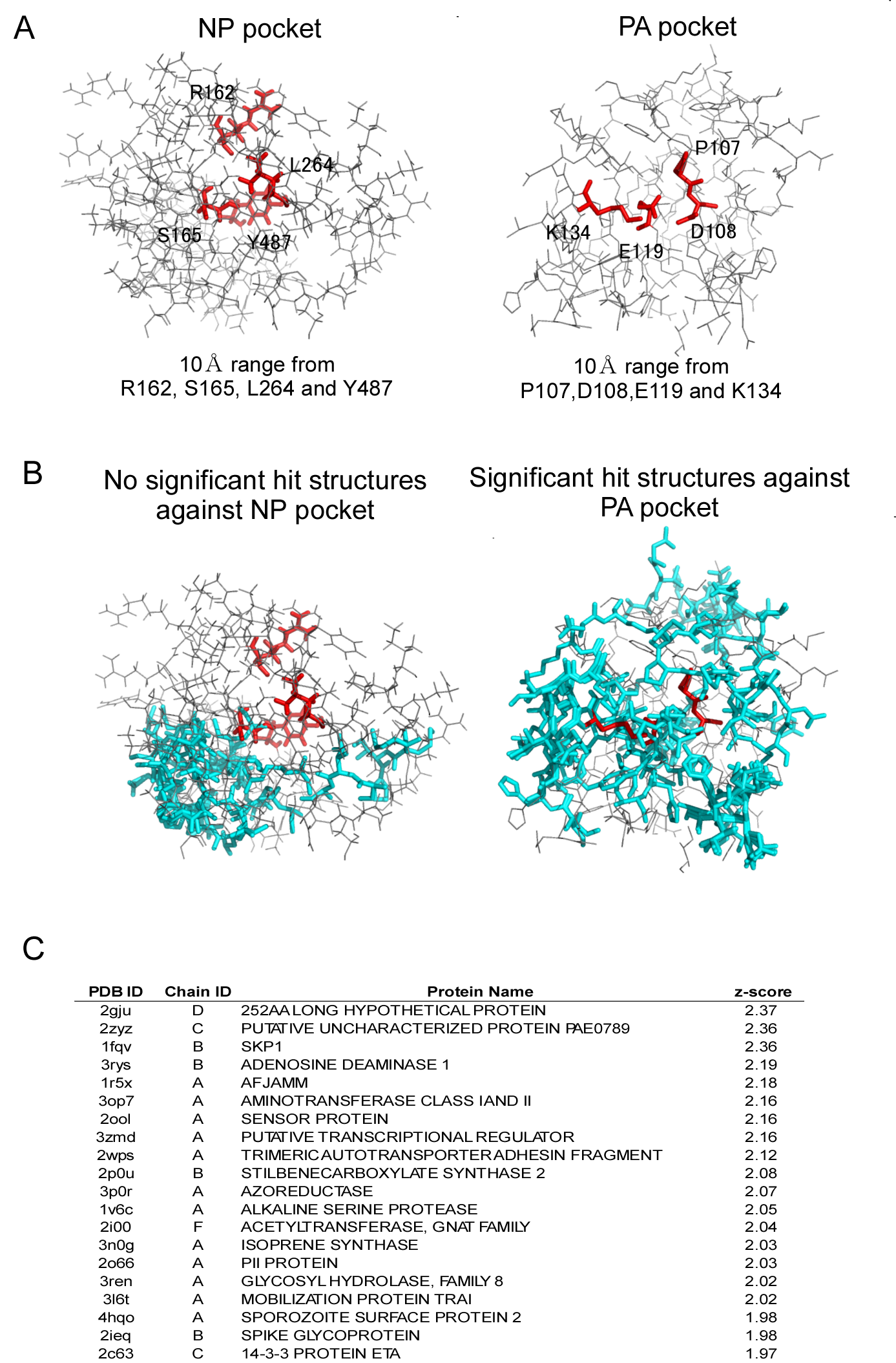

Supplement: S10 Fig — (A) The pocket structures of NP and PA were extracted from complete NP (PDB ID: 2IQH) and PA (PDB ID: 4E5E) structures by removing residues within 10 Å of the small pocket (R162, S165, L264, and Y487) and the endonuclease motif (P107, D108, E119, and K134), respectively. (B) The pocket structures of NP and PA (red) were compared with other potential pocket structures identified from the database (blue). (C) Significant hits (Z-score>1.96) against the PA pocket are shown together with the PDB ID, Chain ID, and Z-score. (TIF) [file ppat.1005062.s010.tif]

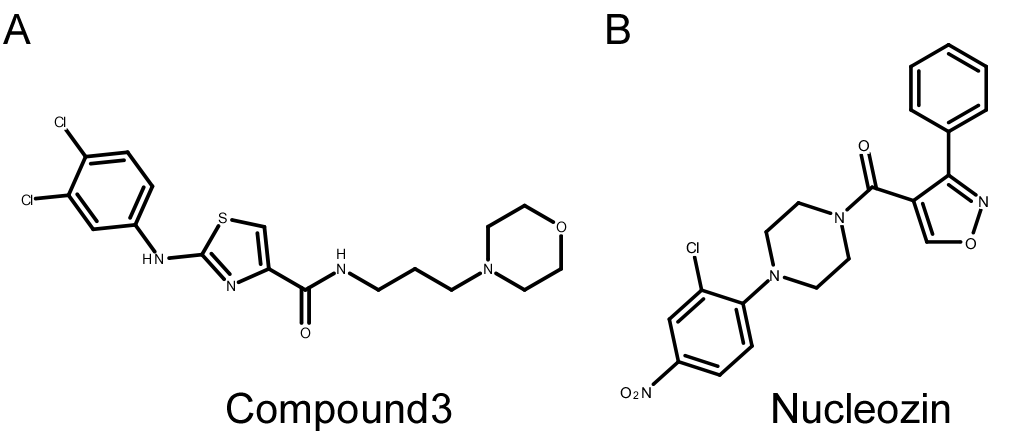

Supplement: S11 Fig — The structure of compound 3 (A) and nucleozin (B) are indicated. Compound 3 inhibits formation of the NP trimer by disrupting the salt bridge between E339 and R416 within the NP tail loop binding pocket [23] and nucleozin inhibits the formation of higher-order NP oligomers by cross-linking two NP molecules [24,25]. (TIF) [file ppat.1005062.s011.tif]

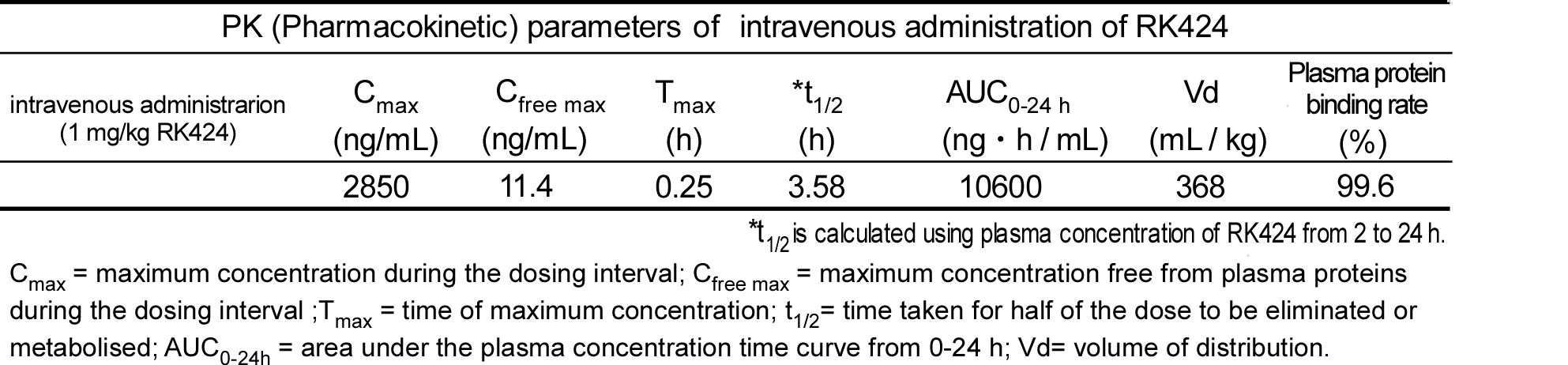

Supplement: S1 Table — PK parameters (Cmax, Cfree max, Tmax, AUC0–24 h, t1/2, and Vd) were calculated using Winnolin Ver.6.1 and different plasma concentrations of RK424. Plasma protein binding was also measured by UPLC. (TIF) [file ppat.1005062.s012.tif]

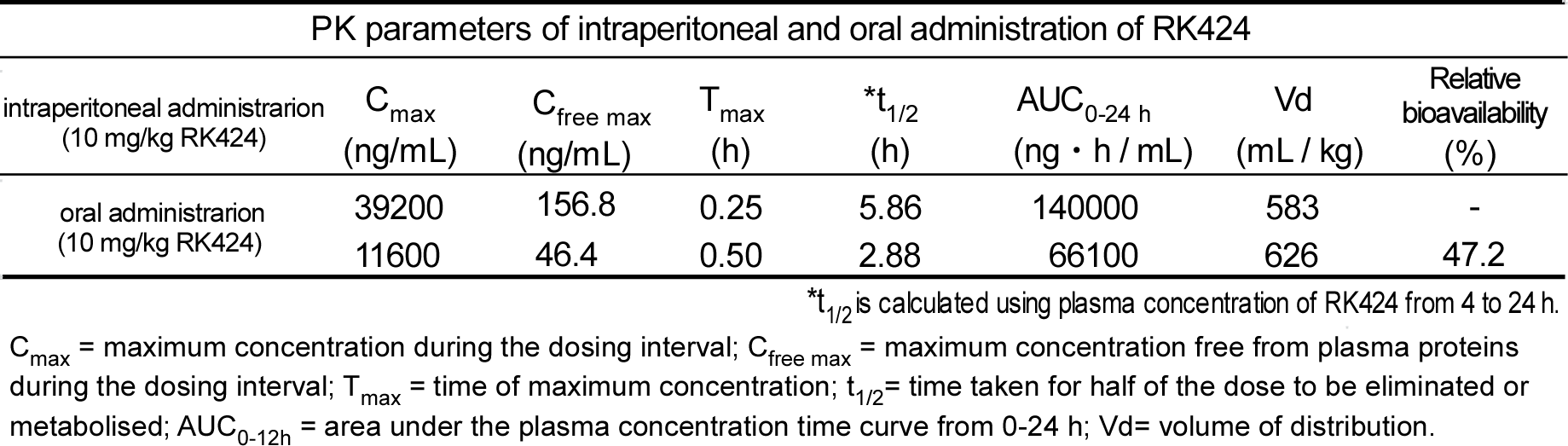

Supplement: S2 Table — PK parameters (Cmax, Cfree max, Tmax, AUC0–24 h, t1/2, and Vd) were calculated using Winnolin Ver.6.1 and different plasma concentrations of RK424. Relative bioavailability was also calculated using intraperitoneal and oral AUC0–24 h. (TIF) [file ppat.1005062.s013.tif]

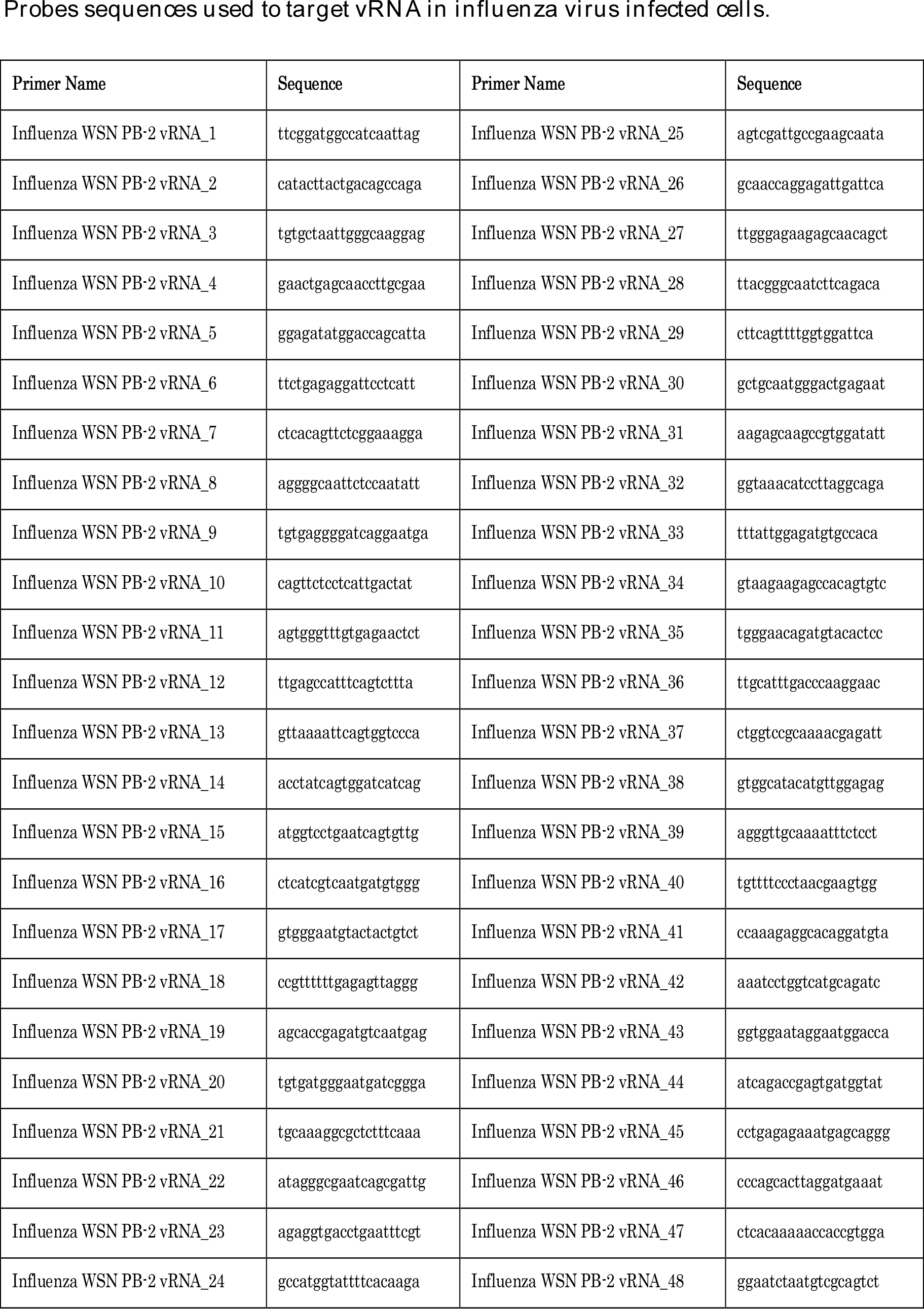

Supplement: S3 Table — The probes were designed by STELLARIS RNA FISH PROBE DESIGNER and purchased from Biosearch Technologies, Inc. (TIF) [file ppat.1005062.s014.tif]
